# Supplementary material for: Retina-derived signals control pace of neurogenesis in visual brain areas but not circuit assembly
Source: Nat Commun. 2023 Sep 27;14:6020. doi: 10.1038/s41467-023-40749-1 (PMC10533834; doi:10.1038/s41467-023-40749-1)
Supplement: Supplementary file 1 — Supplementary Information [file 41467_2023_40749_MOESM1_ESM.pdf]

Supplementary Figure 1

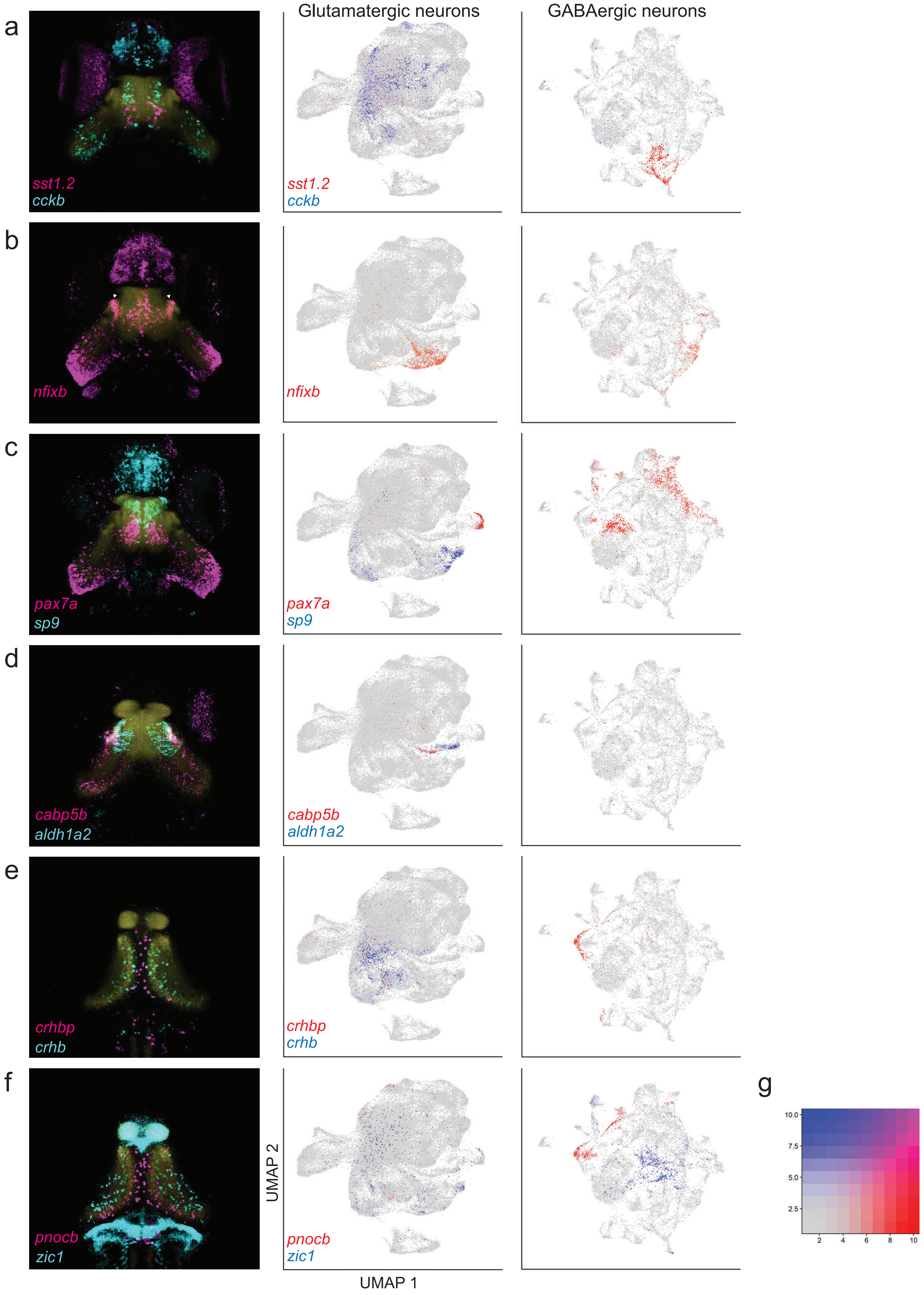

Supplementary Fig. 1: HCR-FISH uncovers the molecular architecture of central visual areas (additional data).

Additional HCR-FISH in-situ stains. (a-f) Substack maximum z-projections of registered HCR-FISH stains. From top to bottom (left): (a-c) Selected thalamic markers: *sst1.2, cckb, nfixb, sp9*. (b-d) Selected pretectal markers: *nfixb* (white arrows point to pretectal area M1), *pax7a, cabp5b, aldh1a2*. (e-f) Selected tectal markers: *crhbp, crhb, pnocb, zic1*. Markers *crhbp* and *crhb* show an additional distinct thalamic population as shown in (Fig. 2). Alongside each stain are UMAPs same as in (Fig. 1), showing where the markers are expressed in glutamatergic clusters (middle) and GABAergic clusters (right). Scale is the same as in (Fig. 2b). For all stains at least three larvae were imaged. Additional stains and anatomical annotations are available in the zebrafish brain atlas[32] at http://mapzebrain.org. (g) Gene expression look-up matrix for combined plots.

Supplementary Figure 2

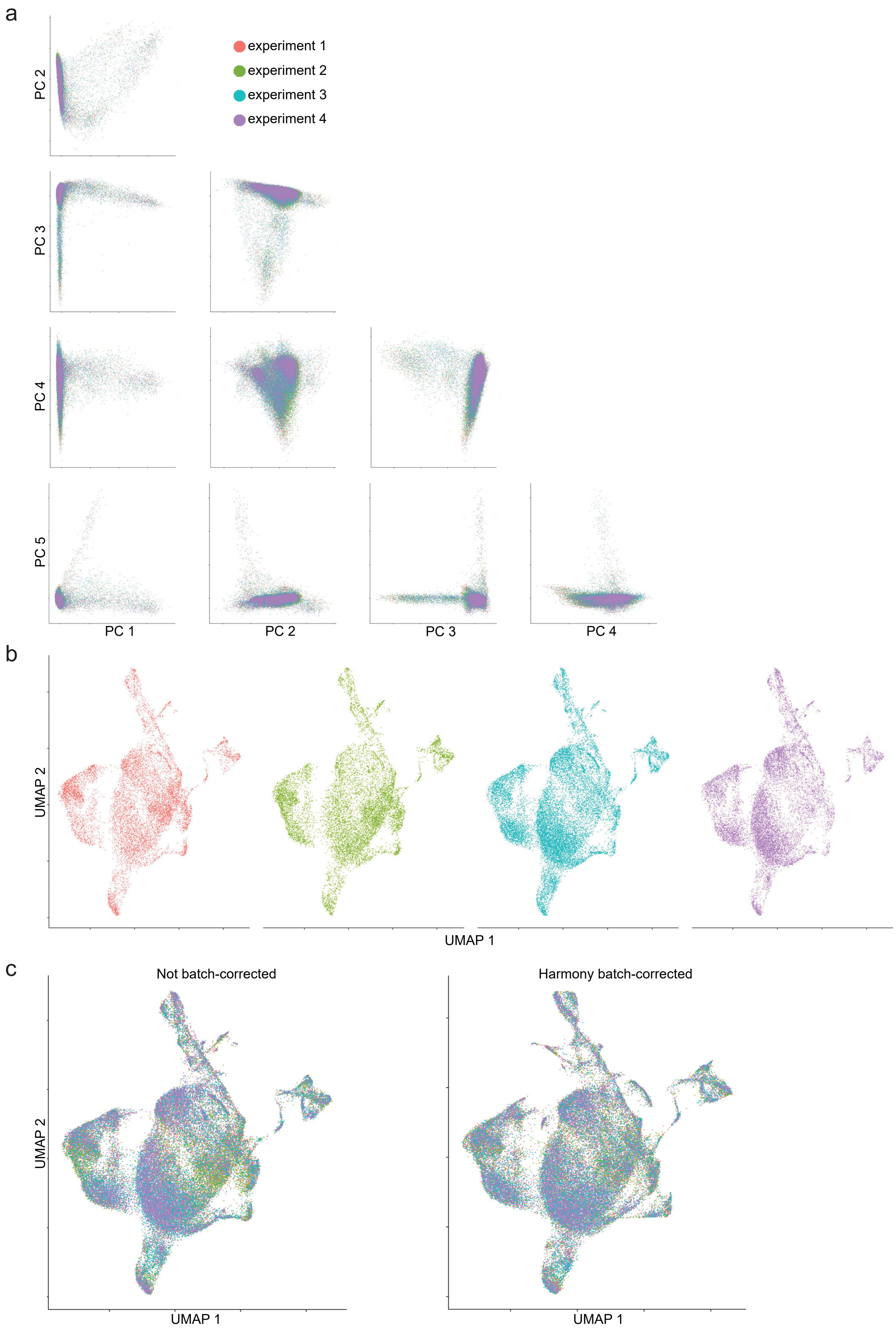

Supplementary Fig. 2: Batch effects on single-cell transcriptomes in WT are limited and can be further corrected by Harmony.

(a) First 5 principal components (PC1 through PC5) of WT cells plotted in order and in all combinations. Color coding and numbering refers to all single-cell samples prepared on the same day. (b) UMAP embedding of WT cells split according to day of sample preparation. Colors are the same as in (a). (c) UMAP embedding of cells before (left) and after (right) batch correction using Harmony. Numbers and colors are the same as in (a) and (b).

Supplementary Figure 3

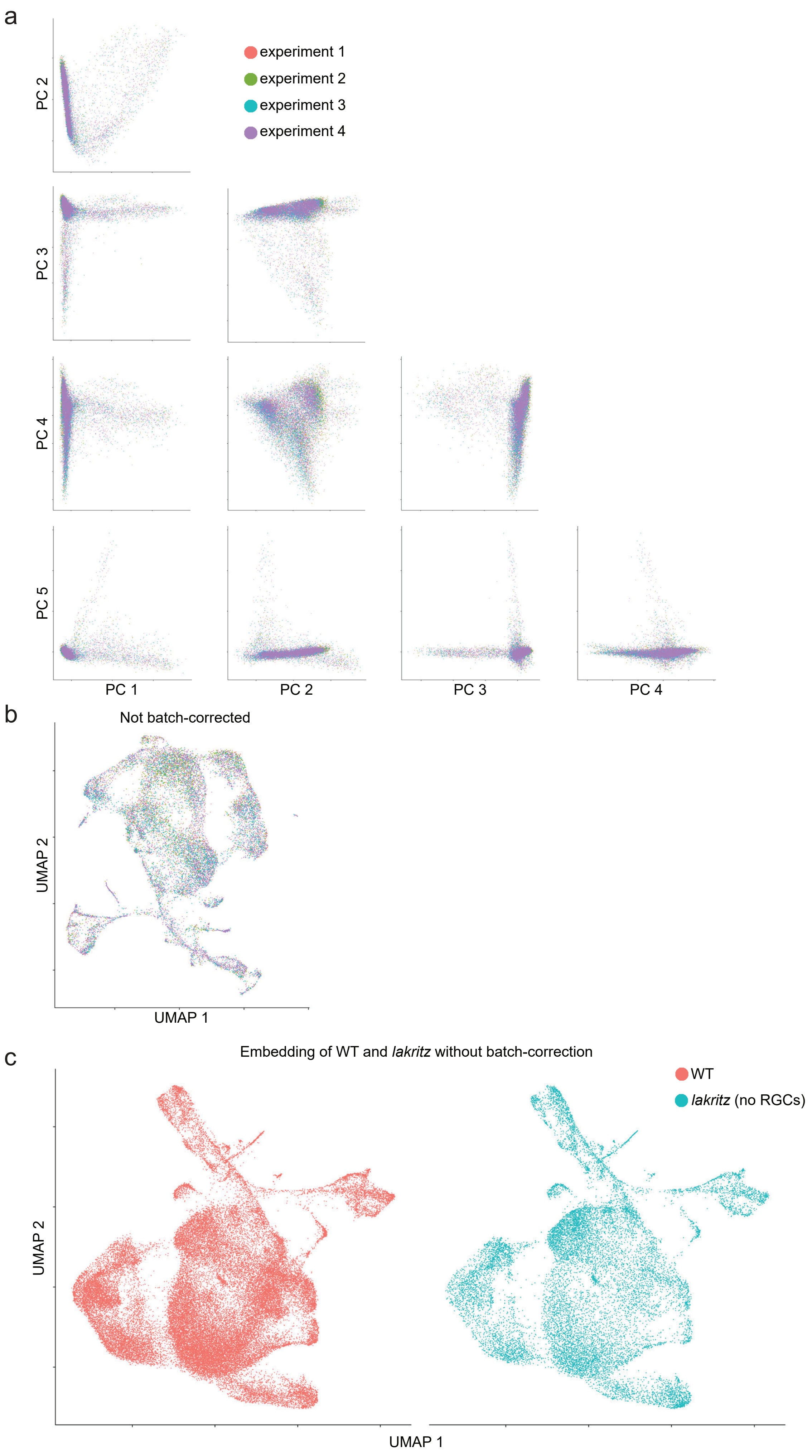

Supplementary Fig. 3: WT and *lakritz* cells can be mapped in the same PC-space with no observable confounding effects.

(a) First 5 principal components of *lakritz* cells (PC1 through PC5) plotted in order and in all combinations. Color coding and numbering refers to all single-cell samples prepared on the same day. (b) UMAP embedding of *lakritz* cells without batch-correction. Numbers and colors are the same as in (a). (c) Embedding of WT and *lakritz* cells in the same space without batch-correction.

Supplementary Figure 4

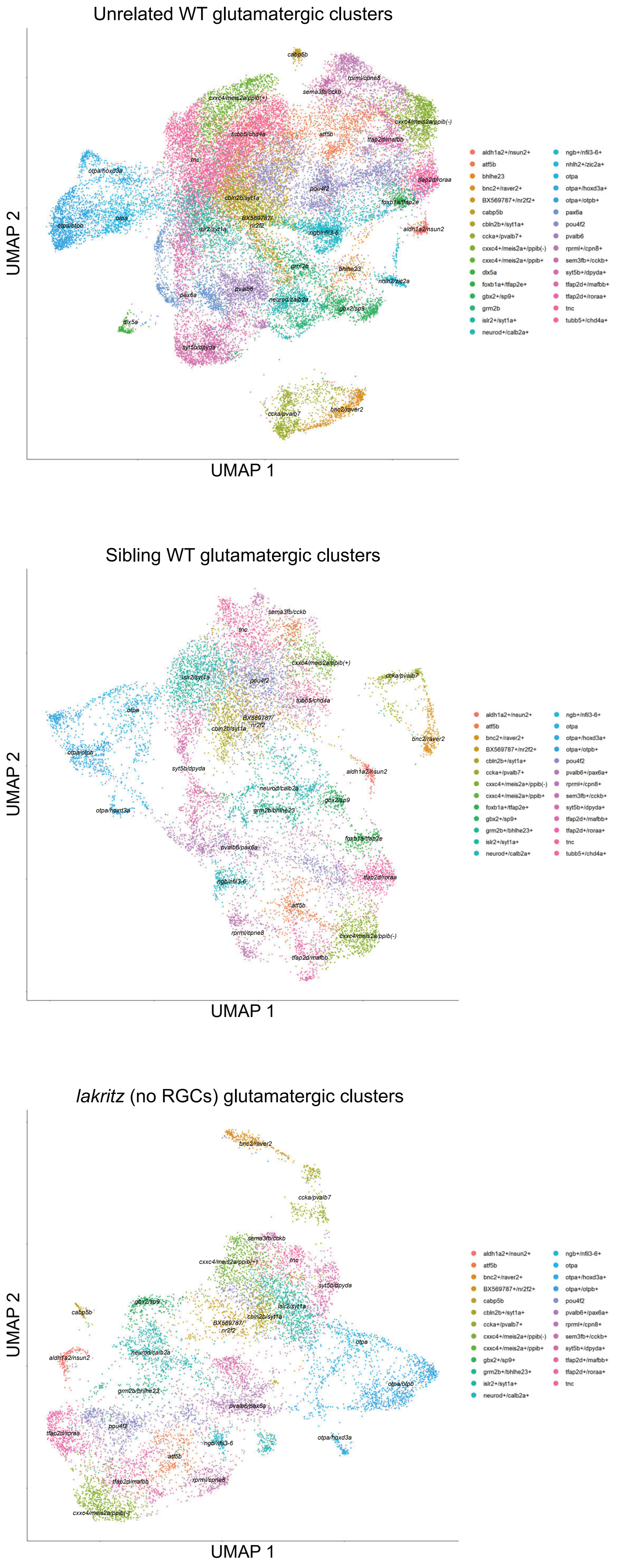

Supplementary Fig. 4: Independent clustering of glutamatergic neurons uncovers similar clusters across genotypes.

UMAP embedding of three different samples (unrelated WT, top; sibling WT, middle; *lakritz*, bottom) after independently processing and clustering each sample. For detailed cluster differences, see Supplementary Fig. 5 and Methods.

Supplementary Figure 5

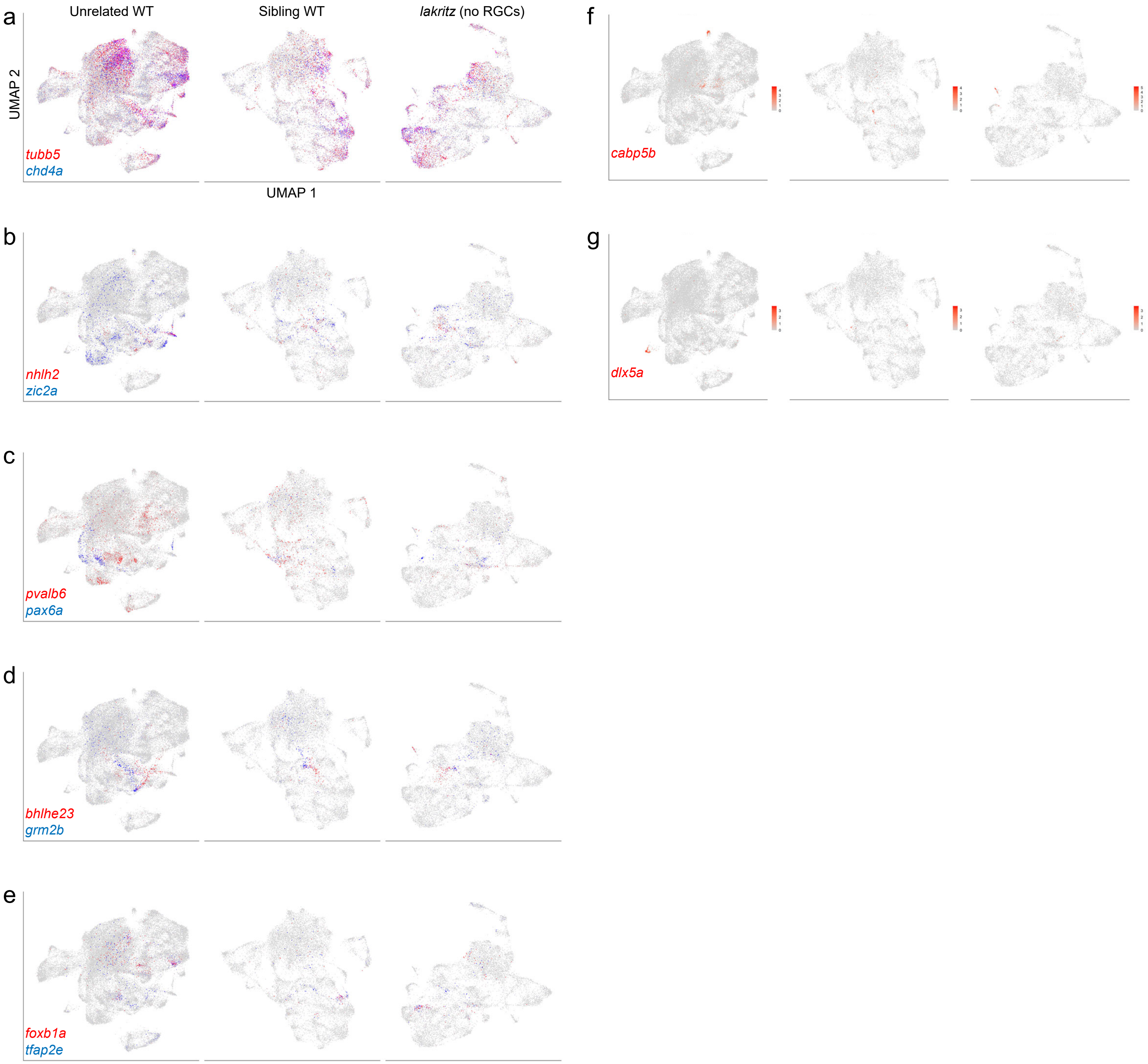

Supplementary Fig. 5: Independent clustering of glutamatergic neurons uncovers similar clusters across genotypes.

Gene expression plots in UMAP-embedded cells after independent processing and clustering of three different genotypes (unrelated WT, sibling WT, *lakritz*). The expression plots show the expression of markers for clusters that are not defined across all three samples. (a-e) Marker genes for clusters which are defined by two markers. Red and blue correspond to the expression of individual markers (unrelated WT, left; sibling WT, middle; *lakritz*, right). (f,g) Marker genes for clusters that are defined by a single marker (unrelated WT, left; sibling WT, middle; *lakritz*, right). For detailed cluster differences, see Methods.

Supplementary Figure 6

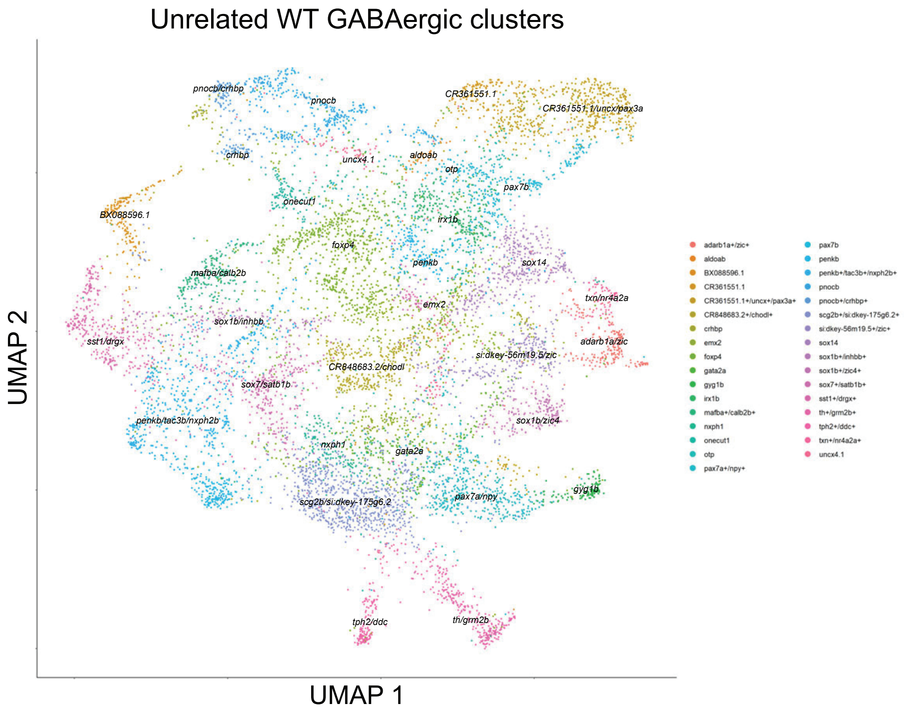

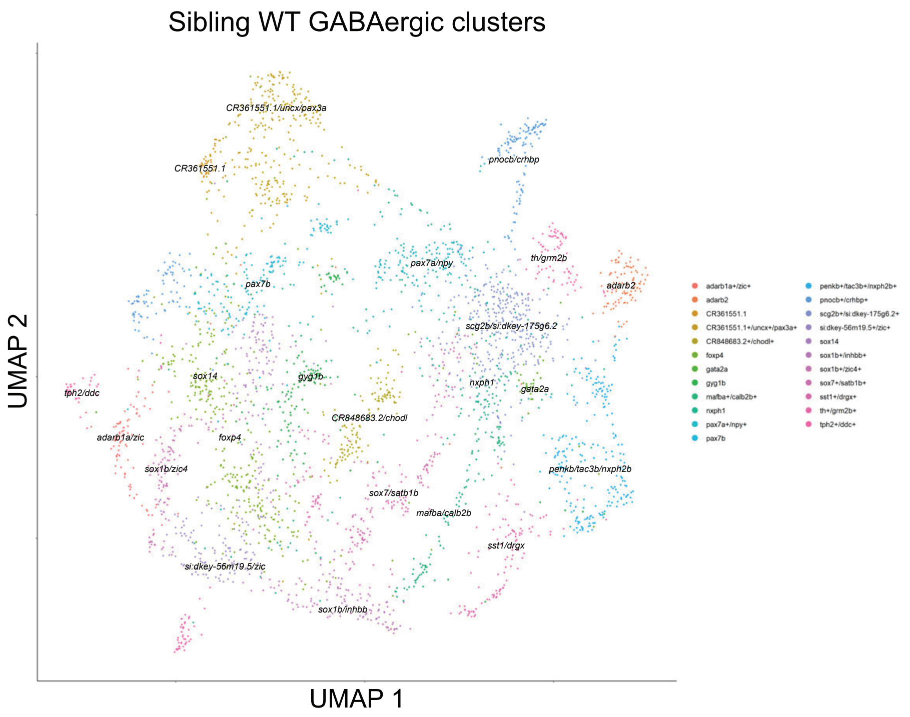

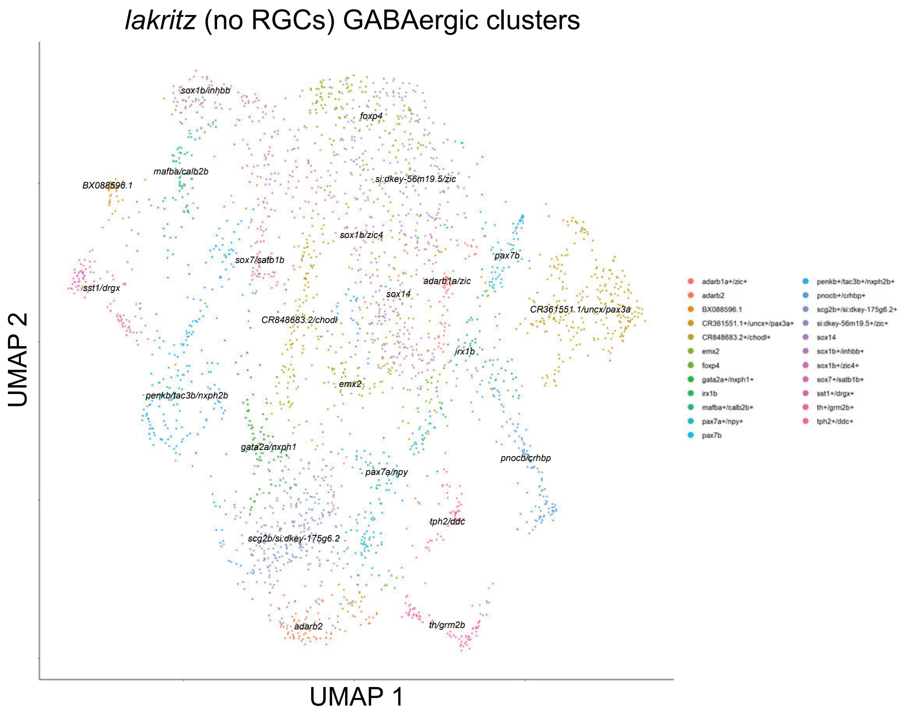

Supplementary Fig. 6: Independent clustering of GABAergic neurons uncovers similar clusters across genotypes.

UMAP embedding of three different samples (unrelated WT, top; sibling WT, middle; *lakritz*, bottom) after independently processing and clustering each sample. For detailed cluster differences, see Supplementary Fig. 7 and methods.

Supplementary Figure 7

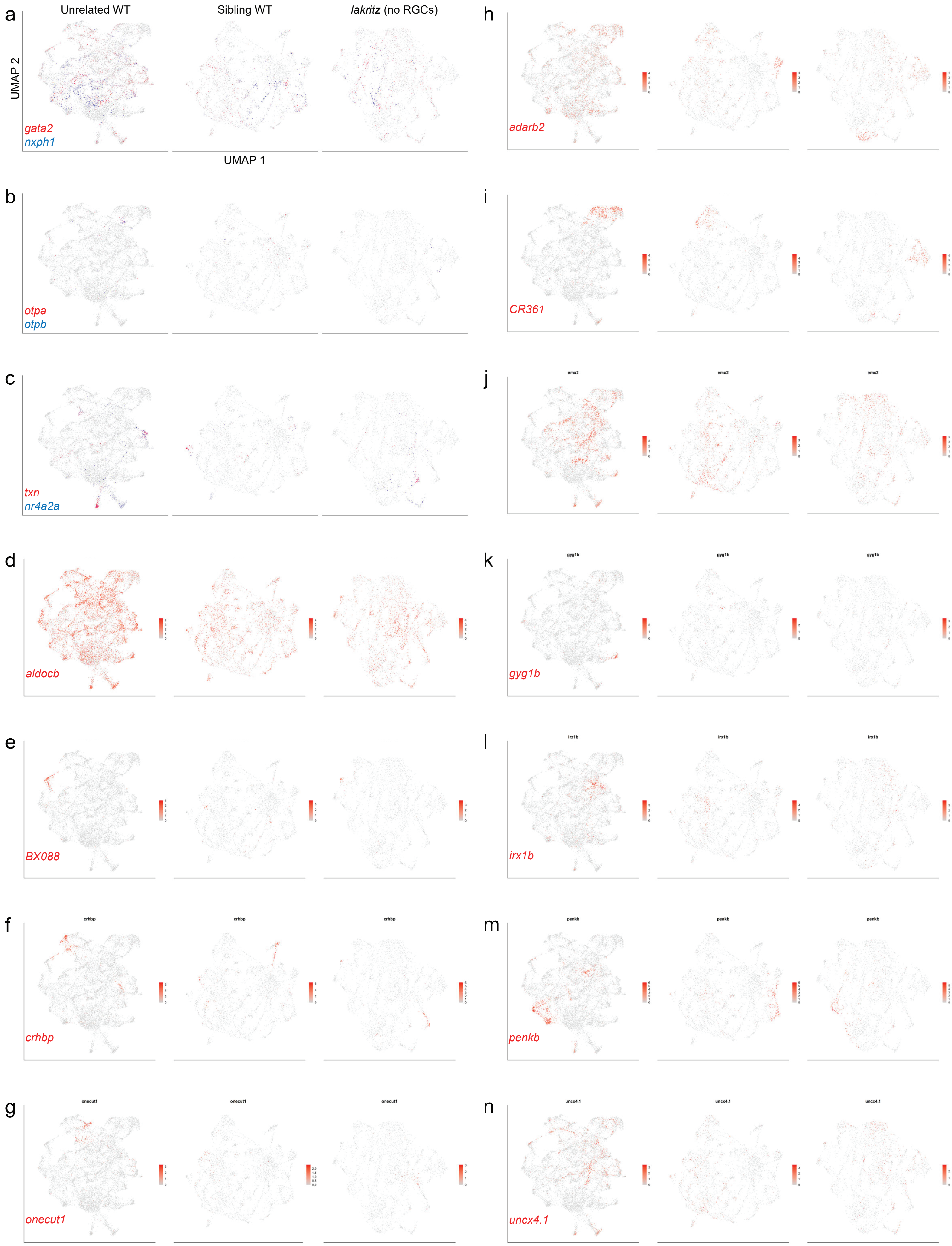

Supplementary Fig. 7: Independent clustering of GABAergic neurons uncovers similar clusters across genotypes.

Gene expression plots in UMAP embedded cells after independent processing and clustering of three different samples (unrelated WT, sibling WT, *lakritz*). The expression plots show the expression of markers for clusters that are not defined across all three samples. (a-c) Marker genes for clusters which are defined by two markers. Red and blue correspond to the expression of individual marker genes (unrelated WT, left; sibling WT, middle; *lakritz*, right). (d-n) Marker genes for clusters which are defined by a single marker (unrelated WT, left; sibling WT, middle; *lakritz*, right). For detailed cluster differences, see Methods.

Supplementary Figure 8

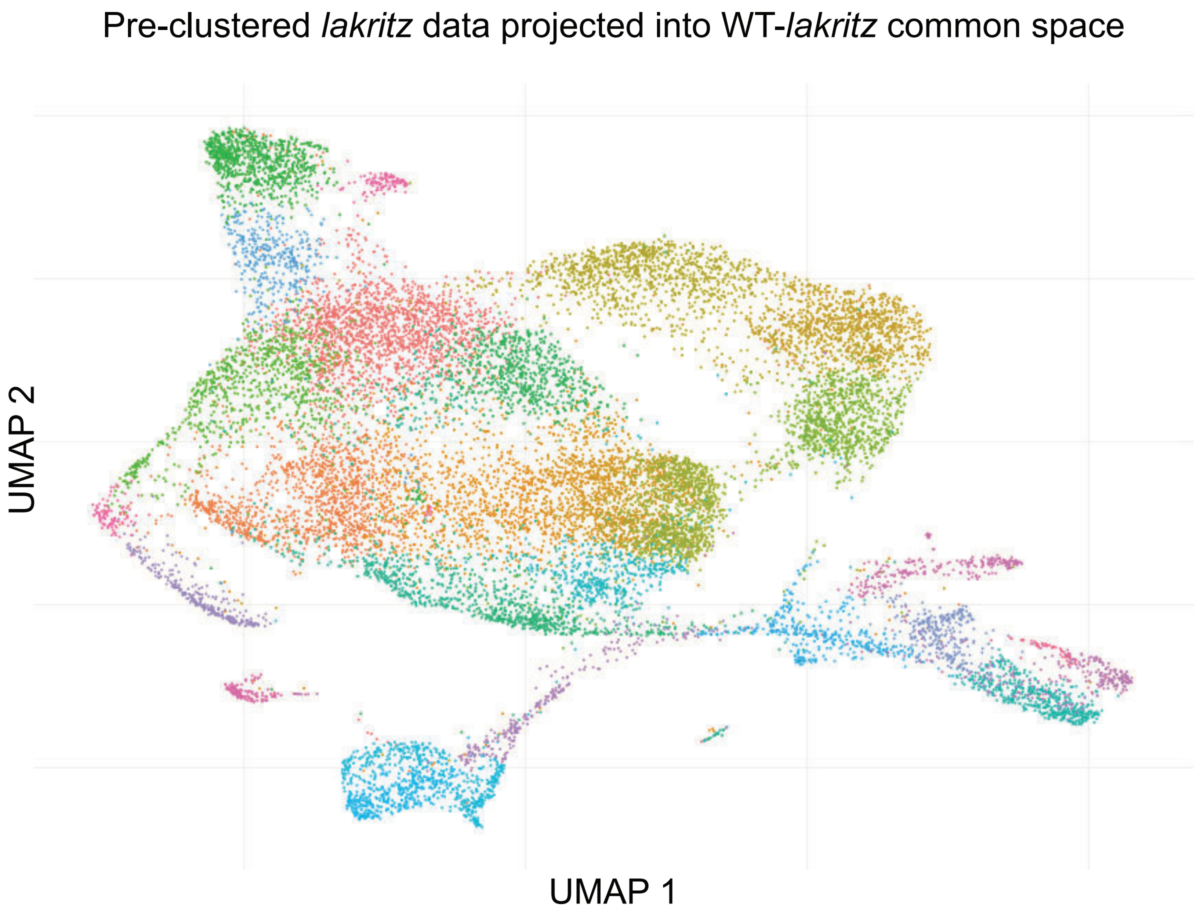

Supplementary Fig. 8: More numerous WT population does not coerce *lakritz* cell-type identities to conform to WT identities.

The *lakritz* cells were pre-clustered, color-coded and embedded with all other cells in common UMAP plot. Mutant clusters retain their neighborhood structure.

Supplementary Figure 9

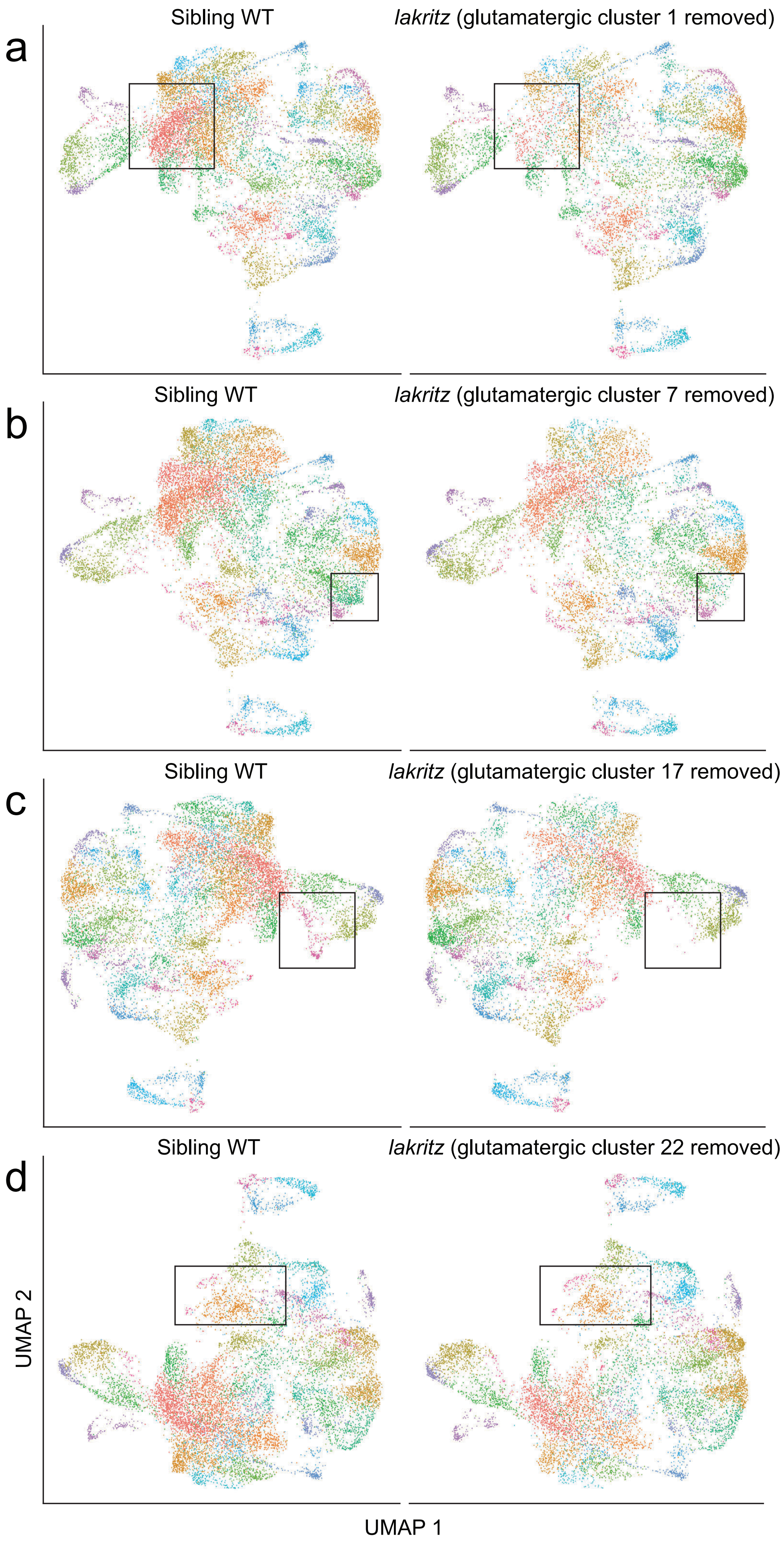

Supplementary Fig. 9: In silico cell-type ablation of a subset of glutamatergic neuronal clusters.

Shown are UMAPs of glutamatergic clusters processed via an in silico cell-type ablation pipeline. (a-c) Examples of three clusters in which a missing cell type from the *lakritz* dataset could be positively identified by visual inspection. (d) Example of a cluster in which identification of the computationally ablated cell type was not evident from visual inspection. Black squares are centered on the position of the ablated cluster.

Supplementary Figure 10

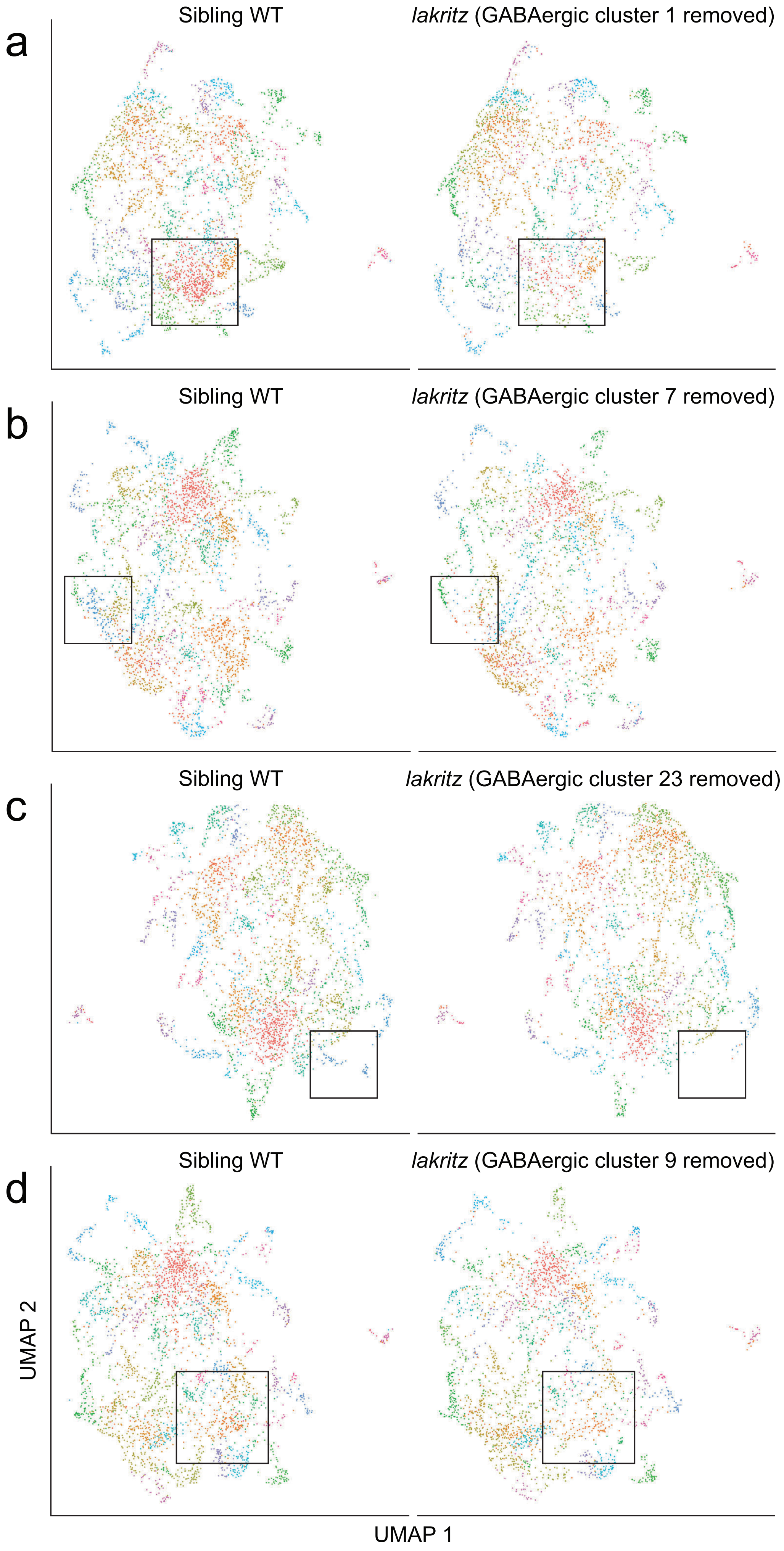

Supplementary Fig. 10: In silico cell-type ablation of a subset of GABAergic neuronal clusters.

Shown are UMAPs of GABAergic clusters processed via an in silico cell-type ablation pipeline. (a-c) Examples of three clusters in which a missing cell type from the *lakritz* dataset could be positively identified by visual inspection. (d) Example of a cluster in which identification of the computationally ablated cell type was not evident from visual inspection. Black squares are centered on the position of the ablated cluster.

Supplementary Figure 11

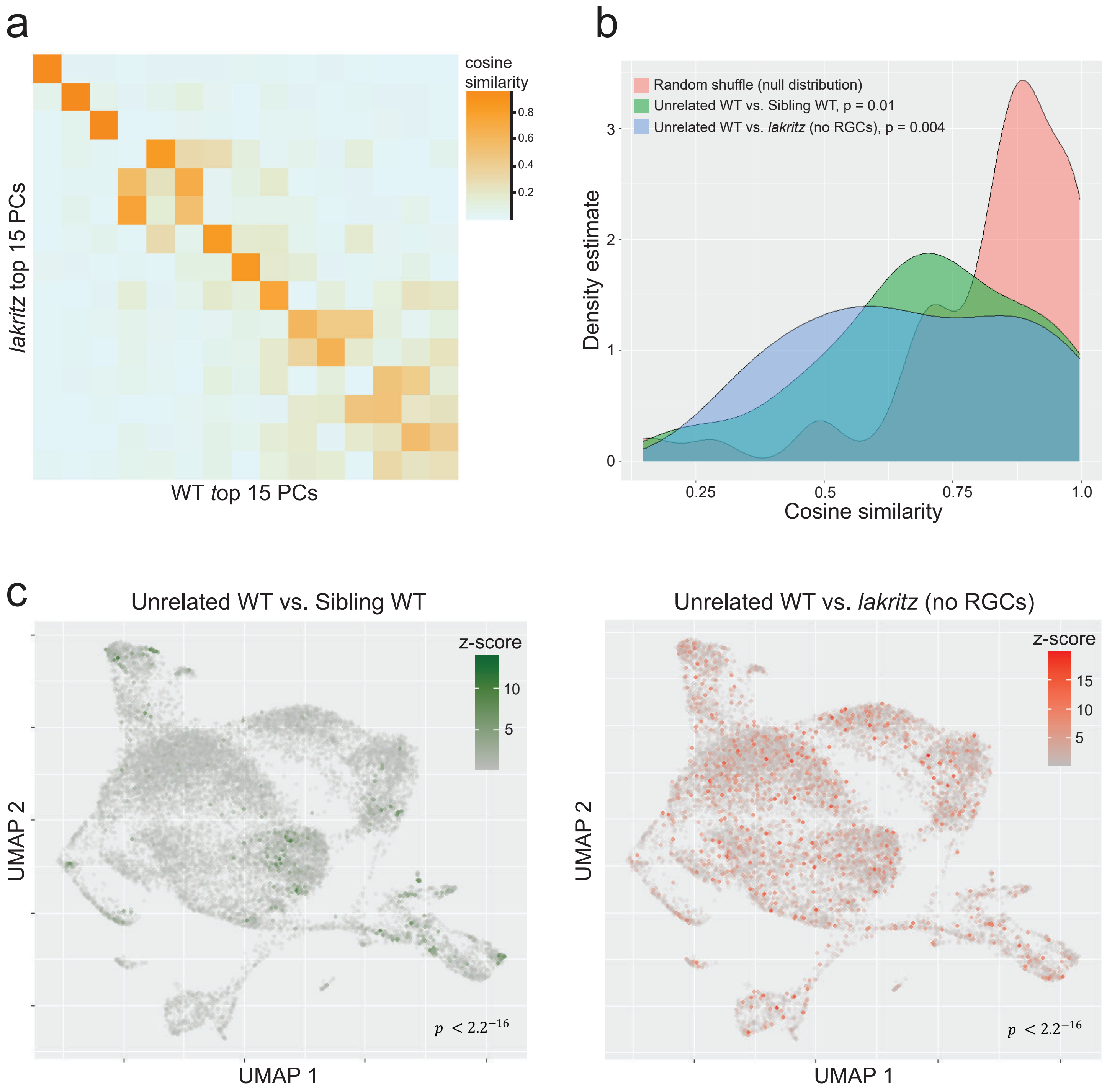

Supplementary Fig. 11: The *lakritz* population shows a global transcriptional drift.

(a) Cosine similarity of the top 15 PCs in the *lakritz* and WT datasets. Color coding shows the most similar PCs across datasets. (b) PC similarity across groups. Red, iterative random shuffle of control cells split into two groups, generating a null distribution. Green, unrelated WT compared with sibling WT. Blue, unrelated WT compared with *lakritz*. The p-values were calculated using a two-sided Wilcoxon signed-rank test. (c) UMAP embedding of all cells. Color-coding shows cells with altered neighborhoods from expected ratio of neighbors from different datasets. Color intensity shows Z-score for severity of neighborhood alteration. The p-values were calculated using a two-sided Wilcoxon signed-rank test comparing either WT (left, green) or *lakritz* (right, red) to a random shuffle distribution.

Supplementary Figure 12

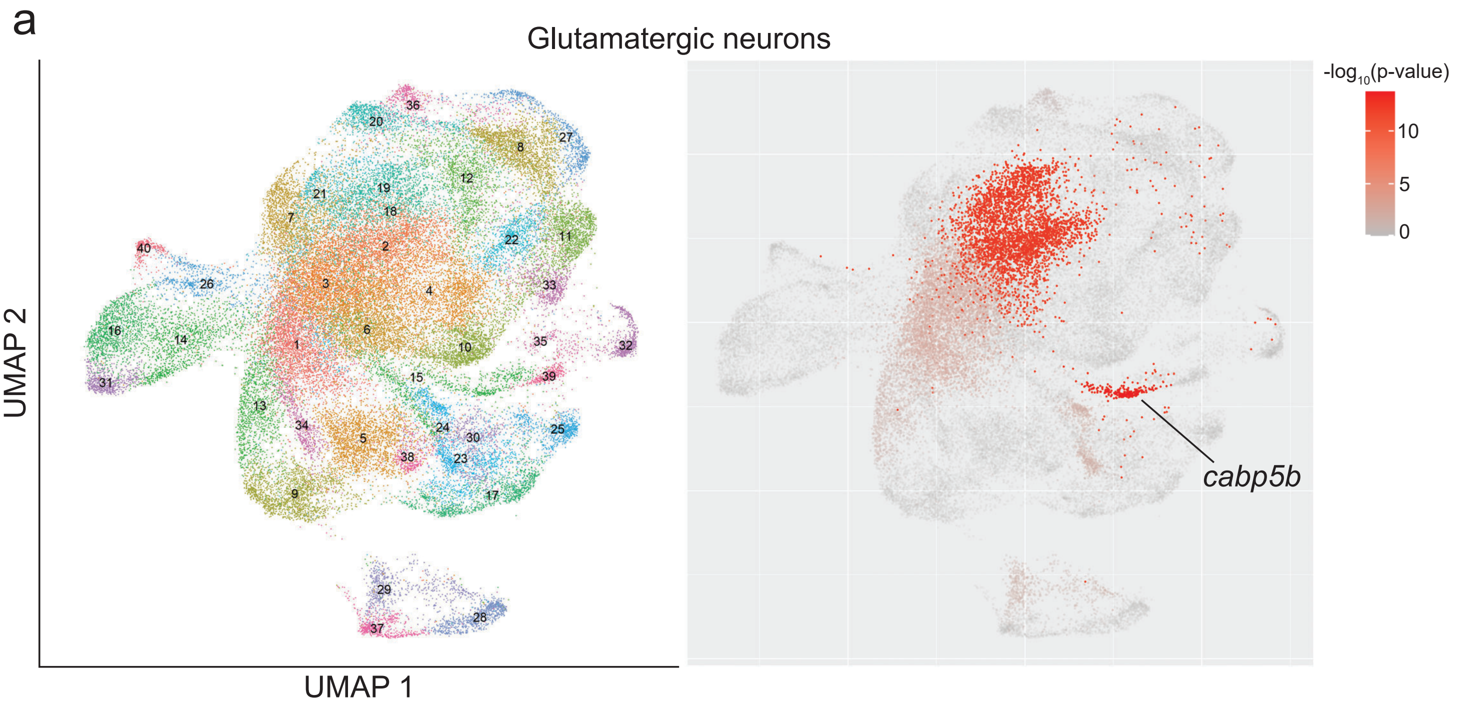

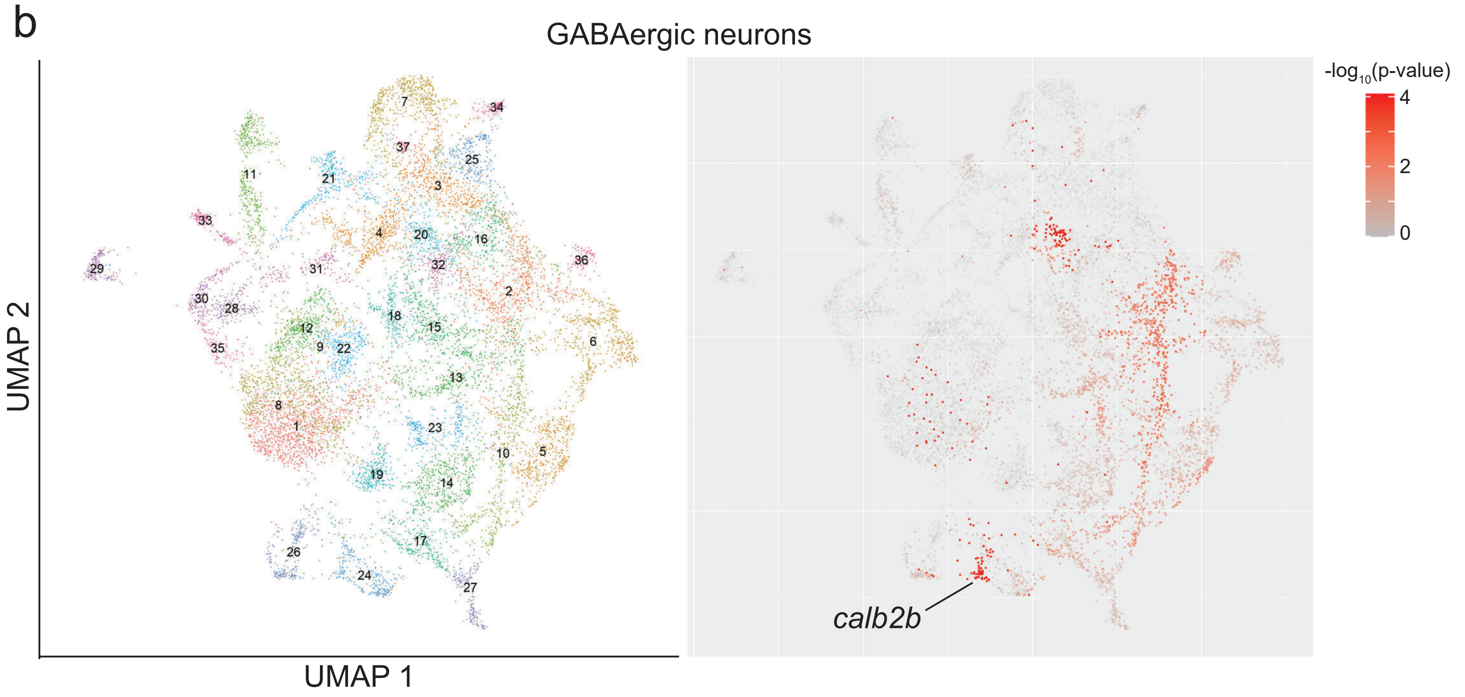

Supplementary Fig. 12: A subset of clusters shows altered transcriptomes within correct fate in absence of RGCs.

(a) Left, UMAP embedding of WT glutamatergic cells color-coded by cluster identity. Right, cells embedded in the same UMAP space as on the left. Cells are color-coded by p-value magnitude calculated from analysis measuring cluster-specific transcriptome changes. Color points to specific clusters more altered than is expected. Line and label show marker of altered cluster. (b) same as in (a), but for GABAergic cells. The p-values were calculated using a two-sided Wilcoxon signed-rank test and corrected for multiple testing using the Bonferroni correction.

Supplementary Figure 13

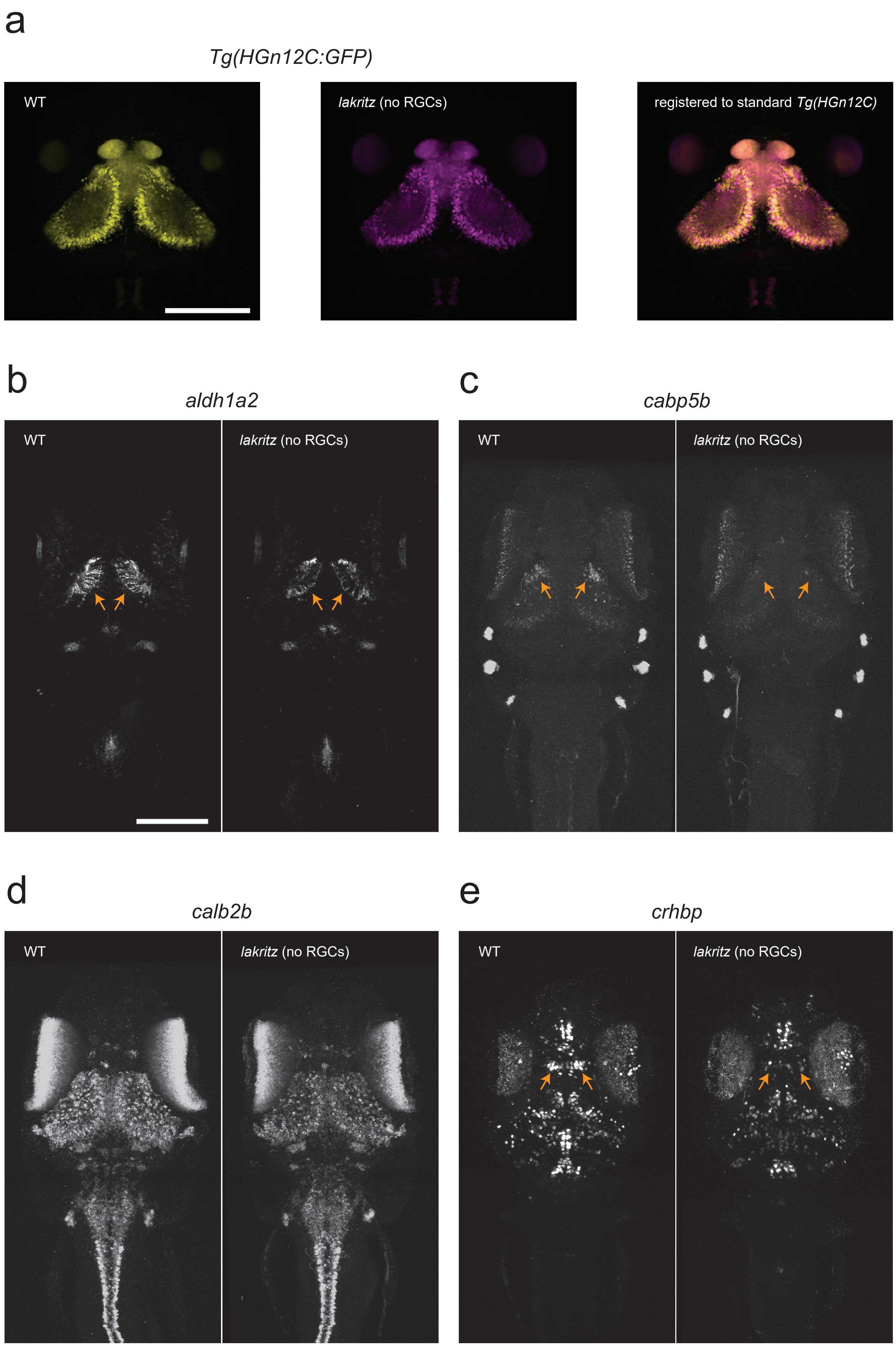

Supplementary Fig. 13: A small subset of markers are locally misexpressed in absence of RGCs

(a) Registration of *Tg(HGn12C)* expression patterns from WT (left, yellow) and *lakritz* (middle, magenta). Right panel shows registered expression patterns to standard *Tg(HGn12C)* pattern (scale bar = 200 µm, applies to all images in (a)). (b-e) Comparative stains between WT (left), and *lakritz* (right). Arrows (b, c, e) show areas where expression is altered in absence of RGCs (scale bar = 200 µm, applies to all images in (b-e)). In (d) the tectum shows broad downregulation. For each stain two samples were imaged.

Supplementary Figure 14

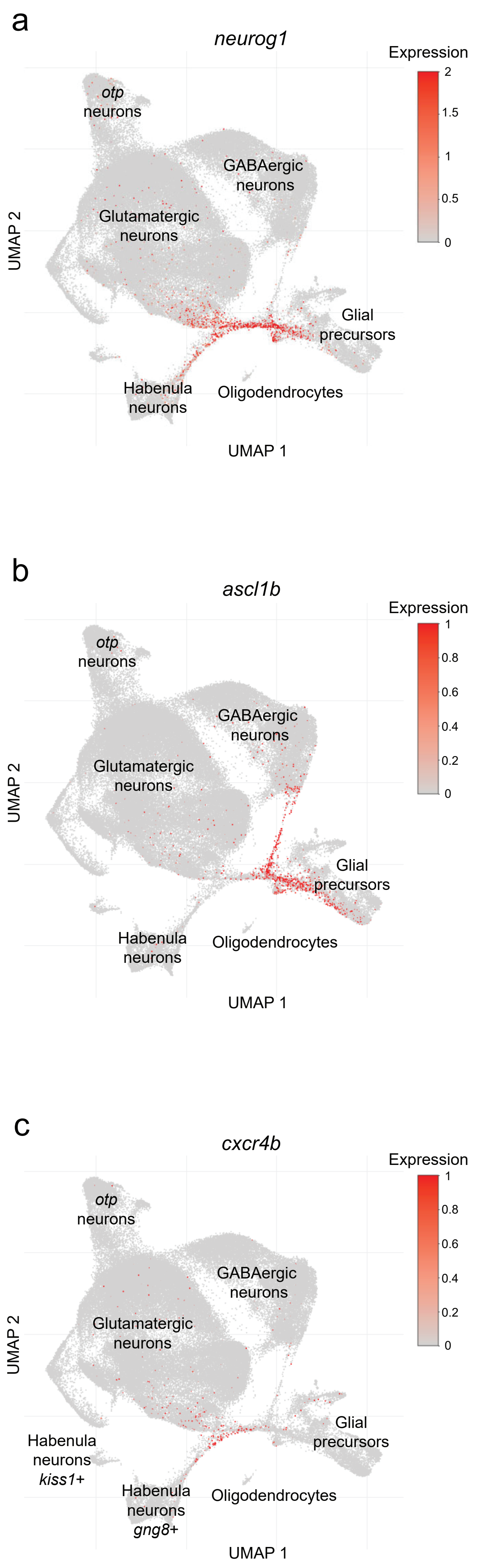

Supplementary Fig. 14: Analysis of precursor marker expression reveals canonical differentiation pathways through transcriptomic space.

(a) UMAP embedding of all cells. Color-coding shows expression levels of *neurog1* in precursor neurons differentiating into glutamatergic neurons (including habenula neurons). (b) UMAP embedding same as in (a). Color-coding shows expression levels of *ascl1b* in precursor neurons differentiating into GABAergic neurons. (c) UMAP embedding same as in a,b. Color-coding shows expression levels of *cxcr4b* in precursor neurons differentiating into habenula neurons.

Supplementary Figure 15

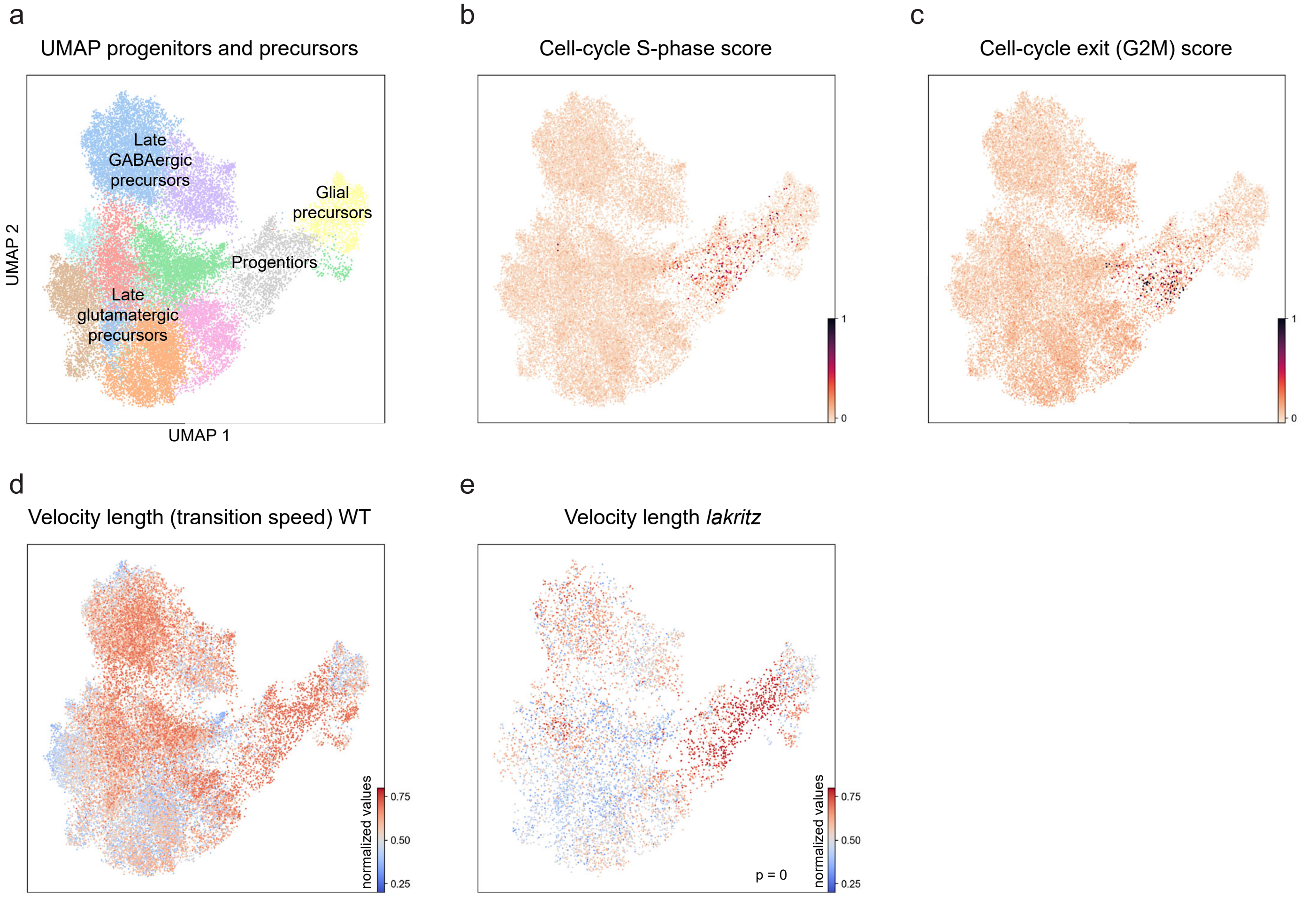

Supplementary Fig. 15: Differentiation trajectories through transcriptomic space are conserved in absence of RGCs but differ in speed (additional data).

(a) UMAP embedding of the same cells as in (Fig. 5). Clusters are color-coded. (b,c) Cells are projected into the same UMAP space as in (a), but color-coded according to their cell-cycle score. (b) Color-coding for score against cell-cycle S-phase genes. (c) Color-coding for score against cell-cycle G2M-phase genes. (d) Expression of glial marker genes *fabp7a*. (e,f) Velocity length inferred from RNA velocity analysis shown in (Fig. 5) for WT (e) or *lakritz* (f). Values were internally normalized to the highest value for visualization. P-value calculated by comparing velocity length between WT and *lakritz* on unnormalized values using a two-sided Wilcoxon signed-rank test.

Supplementary Figure 16

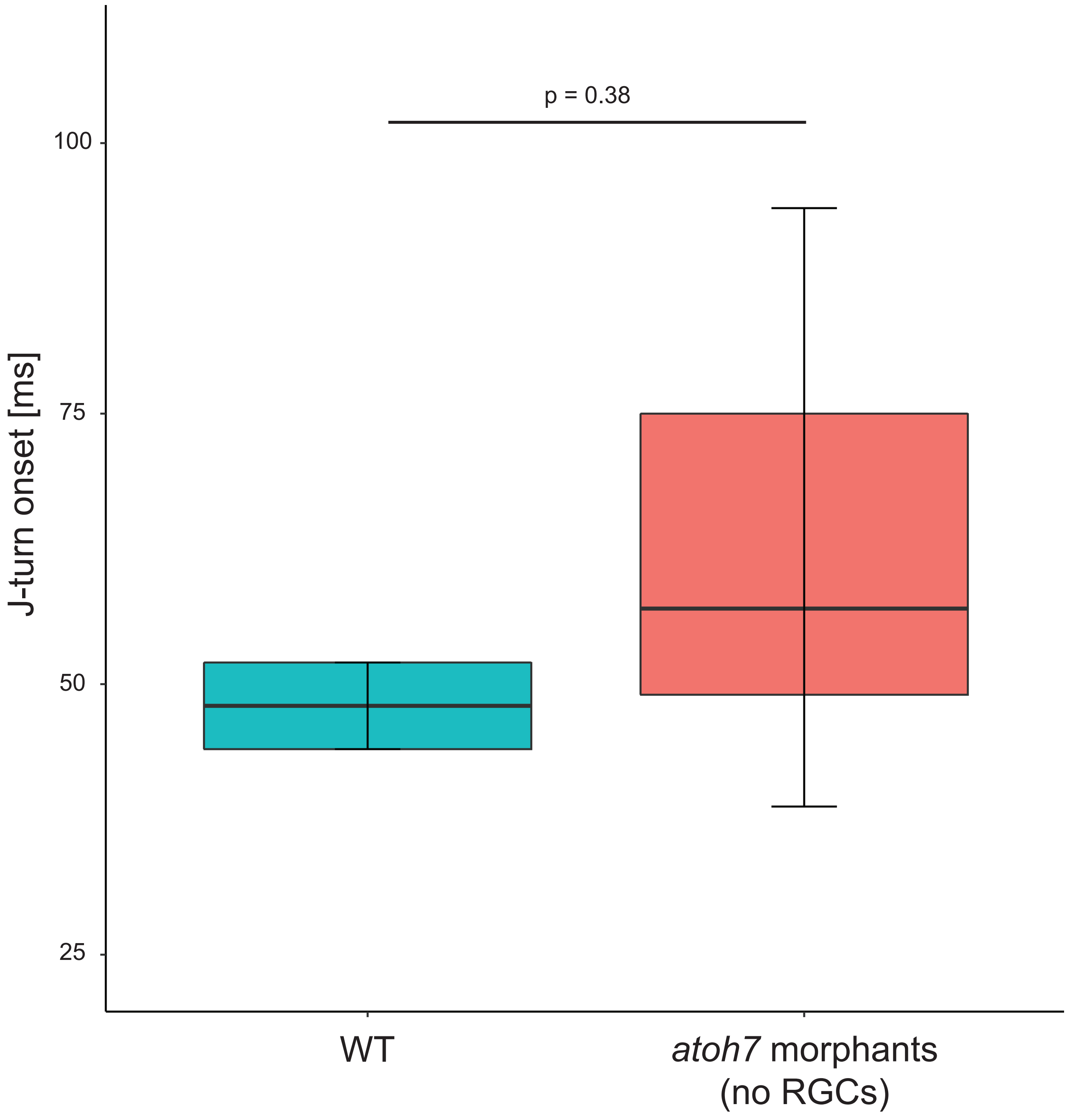

Supplementary Fig. 16: The onsets of optogenetically induced J-turns are more variable in *atoh7* morphants.

Photostimulation of WT and *atoh7* morphants expressing *pvalb6:GAL4* in AF7-connected cells and *UAS:CoChR-tdTomato*. In absence of RGCs, there is a lager variability in the onset of a J-turn after photostimulation. Mean onset is unchanged. The p-values were calculated using a two-sided Wilcoxon signed-rank test (n = 9 independent animals; WT: minima = 44, maxima = 52, center = 48, first Q = 48, third Q = 52, whiskers bounds = 44,52; morphants: minima = 38.67, maxima = 94, center = 57, first Q = 46, third Q = 76, whiskers bounds = 38.67, 94). Source data are provided as a Source Data file.

Supplementary Figure 17

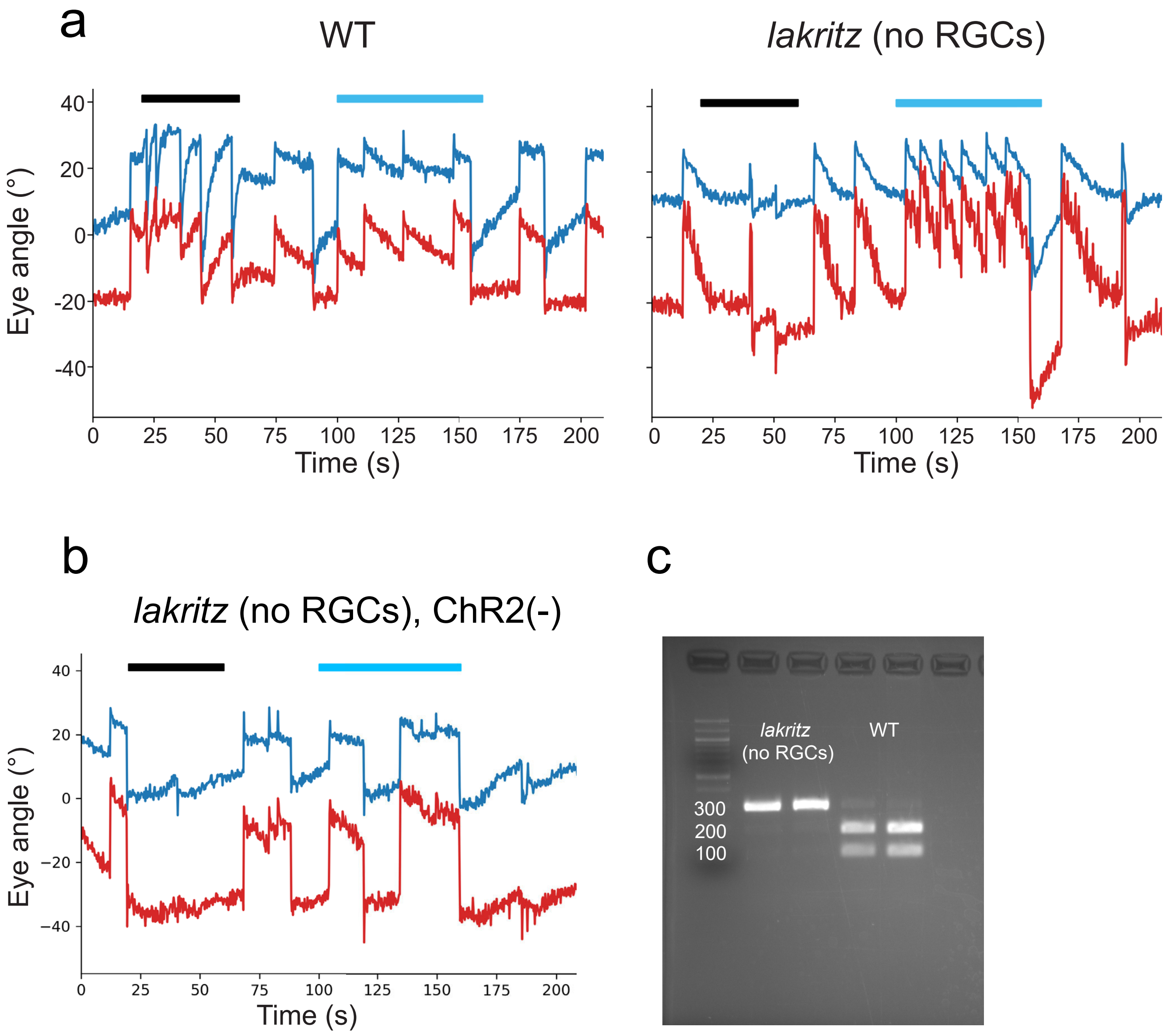

Supplementary Fig. 17: Optogenetic induction of OKR in *lakritz* is robust and specific.

(a) Eye movement traces (blue, left eye; red, right eye) of WT (left) or *lakritz* mutants (right). Black line shows interval of visual stimulation (gratings moving). Cyan line shows interval of optogenetic stimulation. (b) Control *lakritz* (no RGCs) larva same as in (a, right), but lacking ChR2 expression. (c) RFLP analysis confirming a *lakritz* (no RGCs) mutation in larvae selected for pretectal optogenetic stimulation. From left to right: 1,2 lakritz; 3,4 WT controls. DNA ladder is in bp.

Supplementary Figure 18

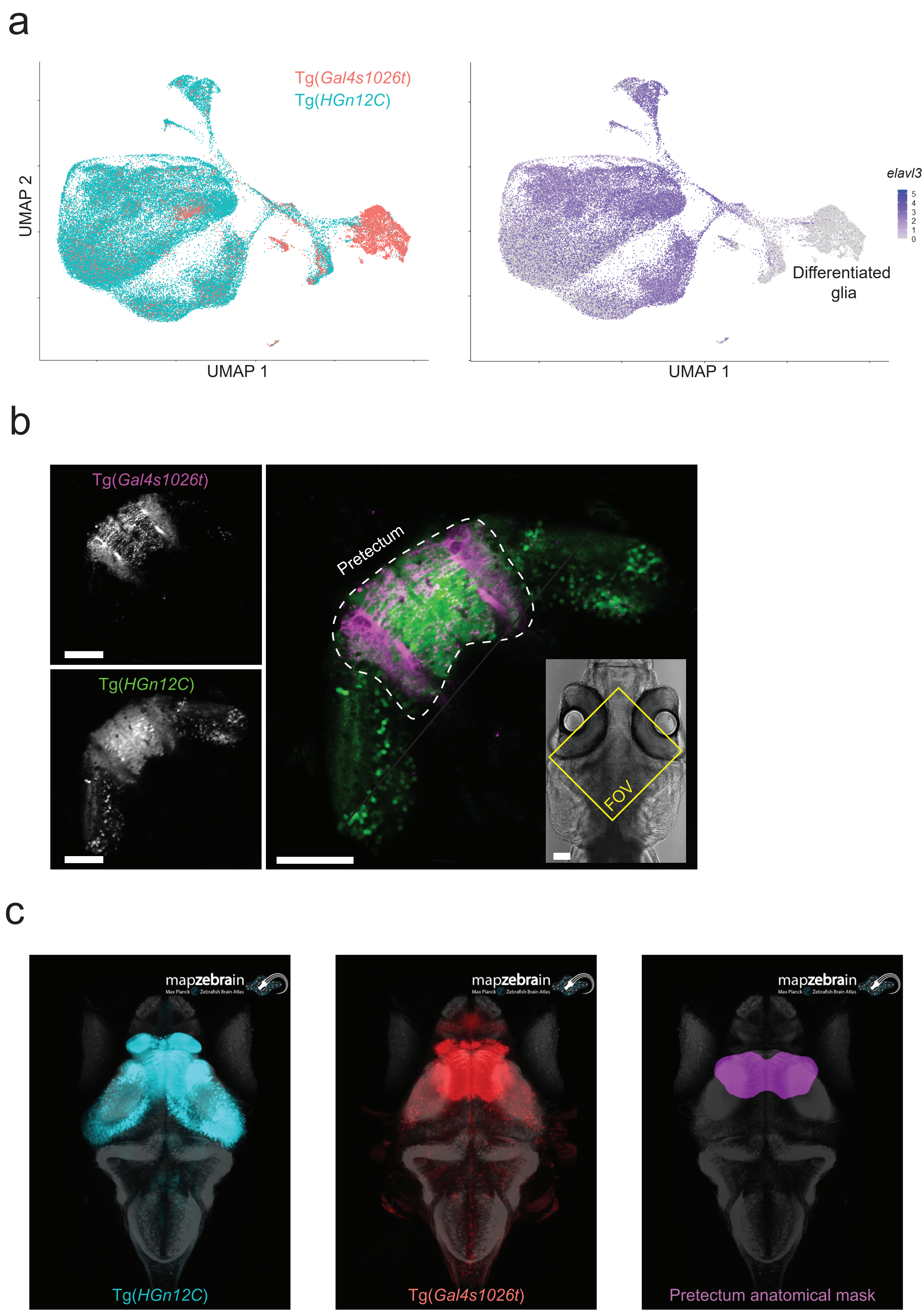

Supplementary Fig. 18: *Tg(HGn12C)* overlaps with *Tg(Gal4s1026t)* expression pattern in pretectal neurons.

(a) UMAP embedding of scRNA-seq results collected from *Tg(Gal4s1026t*; red, left) together with all cells from WT *Tg(HGn12C*; cyan, left). On the right, color coding of *elavl3* expression level across all cells in UMAP space. (b) Pretectal neurons are co-labeled by *Tg(HGn12C*) and Tg(*Gal4s1026t*). A pretectal plane from a single larva is shown (magenta: *Tg(Gal4s1026t*, *UAS:Chr2-mCherry*; green: *Tg(HGn12C:GFP*)). Small panel shows transmitted light and field-of-view (FOV). Scale bars = 100 µm. (c) Registered to standard brain images of transgenic lines *Tg(HGn12C)* (cyan, left) and *Tg(s1026t)* (red, middle). Mask of the pretectum is shown, overlaying the standard brain from http://mapzebrain.org (gray, right).

Table S1

gene	top areas
aldh1a2	pretectum__alar_part, area_postrema, inferior_dorsal_medulla_oblongata_stripe_1, retinal_arborization_field_9
ascl1b	dorsal_thalamus_proper, periventricular_layer, medial_tegmentum_(remaining), pretectum__alar_part
atf5b	periventricular_layer, sac__spv, inferior_dorsal_medulla_oblongata_stripe_5
bhlhe23	dorsal_telencephalon_(pallium), dorsal_thalamus_proper, epiphysis, periventricular_layer, cerebellum
BX088	cerebellum, nucleus_isthmi, olfactory_epithelium, superior_dorsal_medulla_oblongata_stripe_4
cabp5b	stratum_fibrosum_et_griseum_superficiale, sfgs__sgc, pretectum__alar_part
calb1	ventral_telencephalon_(subpallium), pretectum__alar_part, area_postrema, facial_motor_nucleus, vagus_motor_nucleus
calb2a	olfactory_epithelium, cerebellum, pretectum__alar_part, retinal_arborization_field_7, inferior_dorsal_medulla_oblongata_stripe_5
calb2b	stratum_fibrosum_et_griseum_superficiale, inferior_dorsal_medulla_oblongata_stripe_2&3, stratum_opticum, posterior_lateral_line_ganglion, retinal_arborization_field_7
cart2	medial_tegmentum_(remaining), inferior_dorsal_medulla_oblongata_stripe_4, vagal_sensory_lobe, inferior_dorsal_medulla_oblongata_stripe_5
cart3	pretectum__alar_part, olfactory_bulb, epiphysis, nucleus_of_the_medial_longitudinal_fascicle_(pretectum,_basal_part)
ccka	inferior_dorsal_medulla_oblongata_stripe_5, vagus_motor_nucleus, nucleus_isthmi, intermediate_dorsal_medulla_oblongata_stripe_5
cckb	ventral_habenula, periventricular_layer, dorsal_habenula, dorsal_telencephalon_(pallium), olfactory_bulb
chodl	area_postrema, vagus_motor_nucleus, olfactory_bulb, dorsal_telencephalon_(pallium), inferior_dorsal_medulla_oblongata_stripe_5
cort	olfactory_bulb, dorsal_thalamus_proper, ventral_thalamus__alar_part, posterior_tuberculum_(basal_part_of_prethalamus_and_thalamus)
CR361	periventricular_layer, cerebellum
CR848	dorsal_thalamus_proper, pretectum__alar_part, olfactory_epithelium, inferior_dorsal_medulla_oblongata_stripe_5
crhb	dorsal_telencephalon_(pallium), dorsal_thalamus_proper, eminentia_thalami_(remaining), pretectum__alar_part, ventral_thalamus__alar_part
crhbp	ventral_telencephalon_(subpallium), area_postrema, vagus_motor_nucleus, inferior_dorsal_medulla_oblongata_stripe_5
dlx5a	dorsal_telencephalon_(pallium), ventral_telencephalon_(subpallium), eminentia_thalami_(remaining), olfactory_bulb
dopamine_transporter	pretectum__alar_part, posterior_tuberculum_(basal_part_of_prethalamus_and_thalamus), olfactory_bulb, dorsal_thalamus_proper, intermediate_hypothalamus_(remaining)
drgx	inferior_dorsal_medulla_oblongata_stripe_5, intermediate_dorsal_medulla_oblongata_stripe_5, dorsal_habenula, superior_dorsal_medulla_oblongata_stripe_4, intermediate_dorsal_medulla_oblongata_stripe_4
elavl3	dorsal_telencephalon_(pallium), periventricular_layer, dorsal_habenula, ventral_habenula, inferior_dorsal_medulla_oblongata_stripe_4, inferior_dorsal_medulla_oblongata_stripe_5
emx2	periventricular_layer, pretectum__alar_part, eminentia_thalami_(remaining), ventral_entopeduncular_nucleus, torus_longitudinalis, olfactory_epithelium
esrrb	cerebellum, periventricular_layer, pretectum__alar_part
foxb1a	retinal_arborization_field_7, pretectum__alar_part, periventricular_layer, intermediate_hypothalamus_(remaining)
gad1b	stratum_fibrosum_et_griseum_superficiale, inferior_dorsal_medulla_oblongata_stripe_4, inferior_dorsal_medulla_oblongata_stripe_5, stratum_opticum
gbx2	dorsal_thalamus_proper, inferior_dorsal_medulla_oblongata_stripe_2&3, vagal_sensory_lobe, superior_dorsal_medulla_oblongata_stripe_5
gjd2b	stratum_fibrosum_et_griseum_superficiale, sfgs__sgc, stratum_marginale, stratum_opticum
grm2b	dorsal_telencephalon_(pallium), olfactory_bulb, pretectum__alar_part, epiphysis, medial_octavolateralis_nucleus
gyg1b	cerebellum, epiphysis, torus_longitudinalis, dorsal_habenula, stratum_opticum
inhbaa	periventricular_layer, dorsal_thalamus_proper, cerebellum, stratum_marginale, ventral_telencephalon_(subpallium), pretectum__alar_part
insm2	area_postrema, periventricular_layer, intermediate_dorsal_medulla_oblongata_stripe_5, olfactory_bulb, ventral_telencephalon_(subpallium), inferior_dorsal_medulla_oblongata_stripe_5
mafba	intermediate_dorsal_medulla_oblongata_stripe_5, periventricular_layer, intermediate_dorsal_medulla_oblongata_stripe_4, medial_octavolateralis_nucleus, olfactory_bulb
mafbb	periventricular_layer, pretectum__alar_part, olfactory_bulb, olfactory_epithelium
mc5ra	pretectum__alar_part, dorsal_thalamus_proper, periventricular_layer, ventral_telencephalon_(subpallium)
mcm7	periventricular_layer, dorsal_habenula, cerebellum, dorsal_telencephalon_(pallium), torus_longitudinalis
neurog1	dorsal_thalamus_proper, dorsal_habenula
nfil3-6	dorsal_telencephalon_(pallium), periventricular_layer, ventral_habenula, epiphysis
nfixb	periventricular_layer, dorsal_habenula, dorsal_telencephalon_(pallium), cerebellum
ngb	dorsal_habenula, dorsal_telencephalon_(pallium), torus_longitudinalis, olfactory_bulb
npy	periventricular_layer, dorsal_telencephalon_(pallium), pretectum__alar_part, dorsal_thalamus_proper, medial_tegmentum_(remaining), preoptic_region
nsun2	periventricular_layer, epiphysis, torus_longitudinalis
ntn1b	inferior_dorsal_medulla_oblongata_stripe_1, medial_tegmentum_(remaining), olfactory_epithelium
onecut1	ventral_habenula, dorsal_habenula, periventricular_layer, ventral_telencephalon_(subpallium), vagus_motor_nucleus
otpa	intermediate_dorsal_medulla_oblongata_stripe_2&3, superior_dorsal_medulla_oblongata_stripe_2&3, preoptic_region, intermediate_dorsal_medulla_oblongata_stripe_1
pax3a	periventricular_layer, cerebellum, pretectum__alar_part, inferior_dorsal_medulla_oblongata_stripe_4, superior_dorsal_medulla_oblongata_stripe_2&3
pax6a	olfactory_bulb, ventral_thalamus__alar_part, ventral_telencephalon_(subpallium)
pax7a	pretectum__alar_part, periventricular_layer, dorsal_thalamus_proper, cerebellum
pax7b	periventricular_layer, dorsal_habenula, stratum_opticum, pretectum__alar_part, torus_longitudinalis
pcp4l1	cerebellum, epiphysis, olfactory_bulb, superior_dorsal_medulla_oblongata_stripe_4, dorsal_telencephalon_(pallium)
penkb	periventricular_layer, dorsal_telencephalon_(pallium), dorsal_thalamus_proper, vagus_motor_nucleus, posterior_tuberculum_(basal_part_of_prethalamus_and_thalamus), ventral_telencephalon_(subpallium)
pnocb	nucleus_of_the_medial_longitudinal_fascicle_(pretectum,_basal_part), superior_dorsal_medulla_oblongata_stripe_1_(remaining), trochlear_motor_nucleus, medial_tegmentum_(remaining), olfactory_epithelium, inferior_dorsal_medulla_oblong
pou4f2	periventricular_layer, inferior_dorsal_medulla_oblongata_stripe_5
pth2	dorsal_thalamus_proper, periventricular_layer, inferior_dorsal_medulla_oblongata_stripe_5
sema3fb	nucleus_isthmi, periventricular_layer, torus_semicircularis, epiphysis
six3b	ventral_thalamus__alar_part, dorsal_telencephalon_(pallium)
sox14	periventricular_layer, torus_longitudinalis, cerebellum, torus_semicircularis
sox1b	dorsal_telencephalon_(pallium), ventral_telencephalon_(subpallium), olfactory_bulb, periventricular_layer, ventral_habenula
sox7	intermediate_dorsal_medulla_oblongata_stripe_4, olfactory_epithelium, intermediate_dorsal_medulla_oblongata_stripe_5, medial_octavolateralis_nucleus
sp9	dorsal_telencephalon_(pallium), ventral_telencephalon_(subpallium), olfactory_bulb, eminentia_thalami_(remaining)
sst1.2	dorsal_thalamus_proper, inferior_dorsal_medulla_oblongata_stripe_5, inferior_dorsal_medulla_oblongata_stripe_4, vagus_motor_nucleus
tac1	dorsal_telencephalon_(pallium), olfactory_bulb, superior_dorsal_medulla_oblongata_stripe_2&3, vagus_motor_nucleus
tac3b	olfactory_bulb, dorsal_telencephalon_(pallium), dorsal_thalamus_proper, rostral_hypothalamus
tfap2a	periventricular_layer, inferior_dorsal_medulla_oblongata_stripe_4, medial_octavolateralis_nucleus, inferior_dorsal_medulla_oblongata_stripe_5
tfap2b	periventricular_layer, pretectum__alar_part, medial_octavolateralis_nucleus, torus_longitudinalis, inferior_dorsal_medulla_oblongata_stripe_4, inferior_dorsal_medulla_oblongata_stripe_5
tfap2d	periventricular_layer, dorsal_thalamus_proper, torus_semicircularis, nucleus_isthmi, pretectum__alar_part
tfap2e	periventricular_layer, torus_longitudinalis, cerebellum, olfactory_bulb, pretectum__alar_part
th	area_postrema, pretectum__alar_part, vagus_motor_nucleus, vagal_sensory_lobe
tph2	superior_raphe, epiphysis, dorsal_habenula, dorsal_thalamus_proper
txn	olfactory_epithelium, facial_motor_nucleus, superior_raphe
uts1	trochlear_motor_nucleus, nucleus_of_the_medial_longitudinal_fascicle_(pretectum,_basal_part), medial_tegmentum_(remaining), locus_coeruleus
vglut2a	dorsal_habenula, ventral_habenula, epiphysis, inferior_dorsal_medulla_oblongata_stripe_5
zic1	cerebellum, eminentia_thalami_(remaining), dorsal_habenula, torus_longitudinalis, ventral_habenula
zic2a	dorsal_habenula, cerebellum, torus_longitudinalis, dorsal_thalamus_proper, ventral_habenula
zic4	dorsal_habenula, cerebellum, torus_longitudinalis, eminentia_thalami_(remaining), ventral_telencephalon_(subpallium), ventral_habenula

Table S1: Brain areas overlapping with HCR-FISH marker stains.

A table summarizing the top hits for each gene and each brain segment according to current 3D masks in the zebrafish brain atlas at http://mapzebrain.org.

Table S2

	trajectory to:							labels in clusters:				
gene	progenitors	gabaergic clusters	glutamatergic clusters	general habenula	gng8	kiss1		progenitors	gabaergic clusters	glutamatergic clusters	gng8	kiss1
dla	yes	yes	yes	yes	no	no		no	no	no	no	no
hmgb2a	yes	yes	yes	yes	yes	yes		yes	no	yes	no	no
abhd6a	no	yes	yes	no	no	no		no	no	no	no	no
neurog1	no	no	yes	yes	yes	no		no	no	no	no	no
hmga1a	no	yes	yes	yes	yes	yes		no	yes	yes	yes	yes
sox3	no	yes	ambiguous	no	no	no		no	no	no	no	no
notch1a	no	yes	yes	no	no	no		no	no	no	no	no
si:ch73-21g5.7	no	no	no	no	no	no		no	no	no	no	no
hmgb2b	yes	yes	yes	yes	yes	yes		yes	yes	yes	yes	yes
stmn1a	no	yes	yes	yes	no	no		no	ambiguous	ambiguous	no	no
dlb	no	yes	yes	yes	no	no		no	no	no	no	no
notch3	yes	no	no	no	no	no		yes	no	no	no	no
id1	yes	no	no	no	no	no		yes	no	no	no	no
sox11b	no	yes	yes	yes	no	no		no	yes	yes	no	no
si:dkey-151g10.6	yes	yes	yes	yes	yes	yes		yes	yes	yes	yes	yes
ccnd1	yes	no	no	no	no	no		yes	no	no	no	no
msi1	yes	ambiguous	no	yes	yes	no		yes	no	no	ambiguous	no
CU467822.1	yes	yes	yes	no	no	no		yes	yes	yes	no	no
ascl1b.1	no	yes	no	no	no	no		no	no	no	no	no
tpt1	yes	yes	yes	yes	yes	yes		yes	yes	yes	yes	yes
si:ch211-222l21.1	yes	yes	yes	yes	yes	yes		yes	yes	yes	yes	yes
insm1b	no	yes	yes	yes	no	no		no	ambiguous	ambiguous	no	no
rtca	no	yes	yes	no	no	no		no	no	no	no	no
lfng	yes	ambiguous	no	no	no	no		yes	no	no	no	no
ran	yes	yes	yes	yes	yes	yes		yes	yes	yes	yes	yes
BX465834.1	yes	yes	yes	no	no	no		yes	yes	yes	no	no
ebf2	no	no	yes	ambiguous	no	no		no	no	no	no	no
ascl1a	no	yes	no	no	no	no		no	no	no	no	no
rack1	yes	yes	yes	yes	yes	yes		yes	yes	yes	yes	yes
chd7	no	yes	yes	yes	yes	yes		no	no	no	yes	yes
cdkn1ca	no	yes	yes	yes	no	yes		no	no	no	no	yes
zbtb18	no	no	yes	yes	yes	no		no	no	yes	no	no
ddx21	yes	no	no	no	no	no		yes	no	no	no	no
sox19a	yes	no	no	no	no	no		yes	no	no	no	no
hes6	no	yes	yes	yes	no	no		no	ambiguous	ambiguous	no	no
naca	yes	yes	yes	yes	yes	yes		yes	yes	yes	yes	yes
si:dkey-85k7.7	yes	no	no	no	no	no		yes	no	no	no	no
si:dkey-42i9.4	yes	yes	yes	yes	yes	yes		yes	yes	yes	no	no
inavaa	no	ambiguous	ambiguous	no	no	no		no	no	no	no	no
snrpf	no	yes	yes	yes	yes	yes		no	yes	yes	no	no
adh5	yes	yes	yes	yes	yes	yes		ambiguous	ambiguous	yes	no	no
si:ch211-212k18.5	yes	no	no	no	no	no		yes	no	no	no	no
btf3	yes	yes	yes	yes	yes	yes		yes	yes	yes	yes	yes
nfia	ambiguous	yes	yes	no	no	no		no	no	yes	no	no
serbp1a	yes	yes	yes	yes	yes	yes		yes	yes	yes	yes	yes
sb:cb81	yes	no	no	no	no	no		yes	no	no	no	no
fabp7a	yes	no	no	no	no	no		yes	no	no	no	no
msna	yes	no	no	ambiguous	no	no		yes	no	no	no	no
tspan7	no	no	no	no	no	no		no	no	no	no	no
tcf12	yes	ambiguous	ambiguous	yes	ambiguous	no		ambiguous	no	no	no	no
cdk6	no	no	ambiguous	no	no	no		no	no	no	no	no
im:7152348	yes	no	no	yes	yes	no		yes	no	no	yes	no
fgfbp3	yes	yes	no	no	no	no		yes	no	no	no	no
nop58	ambiguous	no	no	no	no	no		no	no	no	no	no
CR751602.2	yes	no	no	no	no	no		yes	no	no	no	no
atp6v0e1	yes	yes	yes	yes	yes	yes		yes	yes	yes	no	no
sox2	yes	ambiguous	no	no	no	no		yes	no	no	no	no
txnipa	yes	no	no	no	no	no		yes	no	no	no	no
ptprz1a	yes	no	no	no	no	no		yes	no	no	no	no
otx2a	ambiguous	ambiguous	no	no	no	no		no	no	no	no	no
zeb2a	yes	ambiguous	no	no	no	no		no	no	no	no	no
snu13b	yes	no	no	no	no	no		no	no	no	no	no
selenoh	yes	no	no	no	no	no		yes	no	no	no	no
sinhcafl.1	yes	yes	yes	yes	no	no		no	yes	yes	no	no
nrarpa	yes	no	no	no	no	no		yes	no	no	no	no
mdka	yes	no	no	no	no	no		yes	no	no	no	no
pax6a	yes	no	no	no	no	no		yes	no	no	no	no
ak2	yes	no	no	no	no	no		yes	no	no	no	no
ahcy	ambiguous	no	no	no	no	no		no	no	no	no	no
tfdp2	no	no	no	no	no	no		no	no	no	no	no
cldn5a	yes	no	no	no	no	no		yes	no	no	no	no
smarcb1b	no	yes	yes	ambiguous	no	no		no	yes	yes	no	no
lima1a	no	ambiguous	no	yes	no	no		no	no	no	no	no
si:dkey-239h2.3	yes	no	no	no	no	no		yes	no	no	no	no
pno1	yes	no	no	no	no	no		yes	no	no	no	no
si:dkey-56m19.5	yes	yes	yes	yes	yes	no		yes	yes	yes	yes	no
nr2f2	yes	yes	yes	yes	yes	no		yes	yes	yes	yes	no
zgc:110796	no	no	no	no	no	no		no	no	no	no	no
tgif1	no	yes	yes	yes	ambiguous	no		no	yes	yes	no	no
npm1a	yes	no	no	no	no	no		yes	no	no	no	no
gng5	ambiguous	no	no	yes	no	no		ambiguous	no	ambiguous	no	no
nop56	yes	ambiguous	ambiguous	ambiguous	no	no		yes	ambiguous	ambiguous	no	no
fgfr3	yes	no	no	no	no	no		yes	no	no	no	no
gtpbp4	yes	yes	yes	yes	no	no		yes	yes	yes	no	no
ybx1	yes	yes	yes	yes	yes	yes		yes	yes	yes	yes	yes
prdx2	yes	yes	yes	yes	yes	yes		yes	yes	yes	yes	ambiguous
CR318588.4	yes	yes	yes	yes	yes	no		yes	yes	yes	yes	no
her6	yes	no	no	no	no	no		yes	no	no	no	no
cebpd	yes	no	no	no	no	no		yes	no	no	no	no
hmgn6	yes	yes	yes	yes	yes	yes		yes	yes	yes	yes	yes
cct2.1	yes	yes	yes	yes	yes	yes		yes	yes	yes	yes	no
nme2b.1	yes	yes	yes	yes	yes	yes		yes	yes	yes	yes	yes
insm1a	no	yes	yes	yes	no	no		no	no	no	no	no
ppiaa	yes	yes	yes	yes	yes	yes		yes	yes	yes	yes	yes
shox2	no	no	yes	no	no	no		no	no	ambiguous	no	no
h2afva	yes	yes	yes	yes	yes	yes		ambiguous	yes	yes	yes	yes
khdrbs1a	yes	yes	yes	yes	yes	yes		yes	yes	yes	yes	yes
rasgef1ba	yes	ambiguous	yes	no	no	no		no	yes	yes	no	no
pou3f2b	no	yes	yes	yes	no	no		no	no	yes	no	no
ddx39ab	yes	yes	yes	yes	no	no		no	yes	yes	no	no
h2afvb	yes	yes	yes	yes	yes	yes		yes	yes	yes	yes	yes
snrpe	yes	yes	yes	yes	yes	yes		no	yes	yes	ambiguous	no
nop10	yes	no	no	no	no	no		yes	no	no	no	no
anp32b	ambiguous	no	no	no	no	no		no	no	no	no	no
lmnb2	no	no	ambiguous	no	no	no		no	no	no	no	no
smc1al	no	yes	yes	yes	no	no		no	yes	yes	no	no
cct3	yes	yes	yes	yes	yes	yes		no	yes	yes	no	no
si:dkey-67c22.2	yes	yes	yes	yes	yes	ambiguous		yes	yes	yes	yes	no
rad21a	yes	yes	yes	yes	yes	yes		no	yes	yes	yes	no
CR848812.1	ambiguous	no	no	no	no	no		no	no	no	no	no
TXN	yes	yes	yes	yes	yes	yes		yes	yes	yes	yes	no

Table S2. Putative differentiation markers.

Table of markers from precursor cluster. Each gene is categorized according to its transient vs. sustained expression pattern in differentiated clusters.

Table S3

gene	age (dpf)	anatomical mask	expression fold change (log2)	adjusted p-value
ascl1b	5	cerebellum ventricle	0.1585	1
ascl1b	5	diencephalon ventricle	0.0329	1
ascl1b	5	tectum midline ventricle	-0.2134	1
ascl1b	5	tectum proliferative ventricle	-0.1663	1
ascl1b	5	telencephalon ventricle	0.1389	0.5772
ascl1b	5	cerebellum	-0.0781	1
ascl1b	5	dorsal thalamus	-0.0415	1
ascl1b	5	pretectum	-0.0399	1
ascl1b	5	ventral thalamus	0.1261	1
ascl1b	5	tectum cell layers	-0.0677	1
ascl1b	5	telencephalon	-0.0068	1
axin2	5	cerebellum ventricle	0.0859	1
axin2	5	diencephalon ventricle	0.0531	1
axin2	5	tectum midline ventricle	-0.0726	1
axin2	5	tectum proliferative ventricle	0.0113	1
axin2	5	telencephalon ventricle	0.0538	1
axin2	5	cerebellum	-0.0652	1
axin2	5	dorsal thalamus	0.0287	1
axin2	5	pretectum	0.0163	1
axin2	5	ventral thalamus	0.162	0.3538
axin2	5	tectum cell layers	-0.1008	1
axin2	5	telencephalon	0.0637	1
cyp19a1b	5	cerebellum ventricle	0.286	1
cyp19a1b	5	diencephalon ventricle	0.055	1
cyp19a1b	5	tectum midline ventricle	0.0697	1
cyp19a1b	5	tectum proliferative ventricle	0.1825	1
cyp19a1b	5	telencephalon ventricle	0.0728	1
cyp19a1b	5	cerebellum	0.2294	1
cyp19a1b	5	dorsal thalamus	0.0211	1
cyp19a1b	5	pretectum	-0.0542	1
cyp19a1b	5	ventral thalamus	0.0811	1
cyp19a1b	5	tectum cell layers	-0.0532	1
cyp19a1b	5	telencephalon	0.1541	0.9052
fabp7a	5	cerebellum ventricle	-0.1872	1
fabp7a	5	diencephalon ventricle	-0.769	2.00E-04
fabp7a	5	tectum midline ventricle	-1.194	1.00E-04
fabp7a	5	tectum proliferative ventricle	-0.7334	3.00E-04
fabp7a	5	telencephalon ventricle	-0.5334	0.0067
fabp7a	5	cerebellum	-0.2775	0.3823
fabp7a	5	dorsal thalamus	-0.9196	1.00E-04
fabp7a	5	pretectum	-1.0535	0
fabp7a	5	ventral thalamus	-0.5531	0.0053
fabp7a	5	tectum cell layers	-0.827	1.00E-04
fabp7a	5	telencephalon	-0.5222	0.0463
gad2	5	cerebellum ventricle	-0.1386	1
gad2	5	diencephalon ventricle	-0.081	1
gad2	5	tectum midline ventricle	-0.5095	0.0047
gad2	5	tectum proliferative ventricle	-0.3374	0.1551
gad2	5	telencephalon ventricle	-0.3047	0.5977
gad2	5	cerebellum	-0.315	0.6888
gad2	5	dorsal thalamus	-0.4146	3.00E-04
gad2	5	pretectum	-0.4055	0.0011
gad2	5	ventral thalamus	-0.3	0.0095
gad2	5	tectum cell layers	-0.7199	0
gad2	5	telencephalon	-0.2001	0.7384
gfap	5	cerebellum ventricle	0.139	1
gfap	5	diencephalon ventricle	-0.1779	1
gfap	5	tectum midline ventricle	0.1191	1
gfap	5	tectum proliferative ventricle	0.0876	1
gfap	5	telencephalon ventricle	-0.002	1
gfap	5	cerebellum	0.0715	1

gfap	5	dorsal thalamus	-0.1381	1
gfap	5	pretectum	-0.1429	1
gfap	5	ventral thalamus	-0.09	1
gfap	5	tectum cell layers	-0.0762	1
gfap	5	telencephalon	0.0928	1
gli1	5	cerebellum ventricle	-0.1353	1
gli1	5	diencephalon ventricle	-0.1236	0.7384
gli1	5	tectum midline ventricle	-0.1285	1
gli1	5	tectum proliferative ventricle	-0.0945	1
gli1	5	telencephalon ventricle	-0.0553	1
gli1	5	cerebellum	-0.135	1
gli1	5	dorsal thalamus	-0.0407	1
gli1	5	pretectum	-0.0429	1
gli1	5	ventral thalamus	0.0686	1
gli1	5	tectum cell layers	-0.0663	1
gli1	5	telencephalon	0.1814	0.4453
her4.1	5	cerebellum ventricle	0.1146	1
her4.1	5	diencephalon ventricle	-0.0469	1
her4.1	5	tectum midline ventricle	-0.0801	1
her4.1	5	tectum proliferative ventricle	-0.0958	1
her4.1	5	telencephalon ventricle	0.1141	1
her4.1	5	cerebellum	0.0366	1
her4.1	5	dorsal thalamus	-0.0245	1
her4.1	5	pretectum	-0.1049	0.3022
her4.1	5	ventral thalamus	0.1551	1
her4.1	5	tectum cell layers	-0.0904	0.791
her4.1	5	telencephalon	0.1429	1
lef1	5	cerebellum ventricle	0.2251	0.4881
lef1	5	diencephalon ventricle	0.0272	1
lef1	5	tectum midline ventricle	0.0194	1
lef1	5	tectum proliferative ventricle	-0.0326	1
lef1	5	telencephalon ventricle	0.1112	1
lef1	5	cerebellum	0.065	1
lef1	5	dorsal thalamus	0.0307	1
lef1	5	pretectum	-0.0494	1
lef1	5	ventral thalamus	0.1408	1
lef1	5	tectum cell layers	-0.0305	1
lef1	5	telencephalon	-0.0054	1
neurod1	5	cerebellum ventricle	-0.532	1
neurod1	5	diencephalon ventricle	-0.462	0.888
neurod1	5	tectum midline ventricle	-0.3831	1
neurod1	5	tectum proliferative ventricle	-0.4937	1
neurod1	5	telencephalon ventricle	-0.3803	1
neurod1	5	cerebellum	-0.5471	1
neurod1	5	dorsal thalamus	0.0438	1
neurod1	5	pretectum	-0.1774	1
neurod1	5	ventral thalamus	0.1316	1
neurod1	5	tectum cell layers	-0.4097	0.8448
neurod1	5	telencephalon	-0.3009	1
neurog1	5	cerebellum ventricle	0.4766	0.0181
neurog1	5	diencephalon ventricle	0.3271	0.9556
neurog1	5	tectum midline ventricle	0.1882	0.2377
neurog1	5	tectum proliferative ventricle	0.3252	0.6699
neurog1	5	telencephalon ventricle	0.5117	0.0957
neurog1	5	cerebellum	0.1569	1
neurog1	5	dorsal thalamus	0.1227	1
neurog1	5	pretectum	0.0683	1
neurog1	5	ventral thalamus	0.2692	0.0789
neurog1	5	tectum cell layers	0.0786	1
neurog1	5	telencephalon	0.0951	1
p27	5	cerebellum ventricle	-0.0438	1
p27	5	diencephalon ventricle	-0.0939	1

p27	5	tectum midline ventricle	-0.0255	1
p27	5	tectum proliferative ventricle	-0.1487	1
p27	5	telencephalon ventricle	-0.1036	1
p27	5	cerebellum	-0.1788	1
p27	5	dorsal thalamus	-0.1158	1
p27	5	pretectum	-0.108	1
p27	5	ventral thalamus	0.0358	1
p27	5	tectum cell layers	-0.1716	1
p27	5	telencephalon	-0.0446	1
pcna	5	cerebellum ventricle	-0.5393	1
pcna	5	diencephalon ventricle	-0.6033	0.1603
pcna	5	tectum midline ventricle	-0.1061	1
pcna	5	tectum proliferative ventricle	-0.5537	1
pcna	5	telencephalon ventricle	-0.4541	1
pcna	5	cerebellum	-0.5237	1
pcna	5	dorsal thalamus	-0.6093	0.0321
pcna	5	pretectum	-0.4451	0.459
pcna	5	ventral thalamus	-0.4499	1
pcna	5	tectum cell layers	-0.4205	1
pcna	5	telencephalon	-0.412	0.994
ptch1	5	cerebellum ventricle	-0.2647	0.9671
ptch1	5	diencephalon ventricle	-0.2874	0.1421
ptch1	5	tectum midline ventricle	-0.515	0.0014
ptch1	5	tectum proliferative ventricle	-0.303	0.3022
ptch1	5	telencephalon ventricle	-0.2598	0.9671
ptch1	5	cerebellum	-0.3407	0.1842
ptch1	5	dorsal thalamus	-0.2853	0.0343
ptch1	5	pretectum	-0.2867	0.0511
ptch1	5	ventral thalamus	-0.2259	0.4128
ptch1	5	tectum cell layers	-0.3439	0.0343
ptch1	5	telencephalon	-0.1739	1
ptch2	5	cerebellum ventricle	0.1294	1
ptch2	5	diencephalon ventricle	-0.1401	1
ptch2	5	tectum midline ventricle	-0.5082	0.0034
ptch2	5	tectum proliferative ventricle	-0.2079	0.4585
ptch2	5	telencephalon ventricle	0.1017	1
ptch2	5	cerebellum	-0.0063	1
ptch2	5	dorsal thalamus	-0.2341	0.0585
ptch2	5	pretectum	-0.2027	0.7206
ptch2	5	ventral thalamus	0.0366	1
ptch2	5	tectum cell layers	-0.2107	0.1271
ptch2	5	telencephalon	0.0369	1
s100b	5	cerebellum ventricle	-0.1168	1
s100b	5	diencephalon ventricle	-0.2923	0.2019
s100b	5	tectum midline ventricle	-0.698	0.0215
s100b	5	tectum proliferative ventricle	-0.189	1
s100b	5	telencephalon ventricle	-0.1815	1
s100b	5	cerebellum	-0.1684	1
s100b	5	dorsal thalamus	-0.1772	1
s100b	5	pretectum	-0.3712	0.2793
s100b	5	ventral thalamus	-0.1702	1
s100b	5	tectum cell layers	-0.2658	0.8902
s100b	5	telencephalon	-0.1436	1
shha	5	cerebellum ventricle	-0.3874	0.0132
shha	5	diencephalon ventricle	-0.1439	0.2369
shha	5	tectum midline ventricle	-0.2421	0.0621
shha	5	tectum proliferative ventricle	-0.4229	6.00E-04
shha	5	telencephalon ventricle	-0.2883	0.0053
shha	5	cerebellum	-0.4697	0.0011
shha	5	dorsal thalamus	-0.2585	0.0095
shha	5	pretectum	-0.3415	1.00E-04
shha	5	ventral thalamus	-0.1234	1

shha	5	tectum cell layers	-0.4445	0
shha	5	telencephalon	-0.2983	0.0118
shhb	5	cerebellum ventricle	-0.1918	0.9556
shhb	5	diencephalon ventricle	-0.0864	0.1271
shhb	5	tectum midline ventricle	-0.1524	0.3913
shhb	5	tectum proliferative ventricle	-0.1255	1
shhb	5	telencephalon ventricle	-0.1789	0.218
shhb	5	cerebellum	-0.2427	0.1271
shhb	5	dorsal thalamus	-0.1359	0.0869
shhb	5	pretectum	-0.1774	0.0227
shhb	5	ventral thalamus	-0.1272	1
shhb	5	tectum cell layers	-0.2363	0.0162
shhb	5	telencephalon	-0.1551	0.4238
sox2	5	cerebellum ventricle	0.0779	1
sox2	5	diencephalon ventricle	0.158	0.6389
sox2	5	tectum midline ventricle	0.1187	1
sox2	5	tectum proliferative ventricle	0.0232	1
sox2	5	telencephalon ventricle	0.1669	1
sox2	5	cerebellum	-0.0011	1
sox2	5	dorsal thalamus	0.0361	1
sox2	5	pretectum	0.0601	1
sox2	5	ventral thalamus	0.2146	0.531
sox2	5	tectum cell layers	0.0719	1
sox2	5	telencephalon	0.1029	1
vglut2a	5	cerebellum ventricle	-0.0058	1
vglut2a	5	diencephalon ventricle	-0.0852	1
vglut2a	5	tectum midline ventricle	-0.1588	1
vglut2a	5	tectum proliferative ventricle	-0.0921	1
vglut2a	5	telencephalon ventricle	0.02	1
vglut2a	5	cerebellum	-0.0088	1
vglut2a	5	dorsal thalamus	-0.2329	0.0309
vglut2a	5	pretectum	-0.2712	0.0204
vglut2a	5	ventral thalamus	-0.1494	1
vglut2a	5	tectum cell layers	-0.4159	0.006
vglut2a	5	telencephalon	-0.0483	1
wnt3	5	cerebellum ventricle	0.0281	1
wnt3	5	diencephalon ventricle	-0.0923	0.6888
wnt3	5	tectum midline ventricle	-0.2943	0.0164
wnt3	5	tectum proliferative ventricle	-0.0469	1
wnt3	5	telencephalon ventricle	0.0581	1
wnt3	5	cerebellum	-0.0766	1
wnt3	5	dorsal thalamus	-0.1236	0.3271
wnt3	5	pretectum	-0.2068	0.0252
wnt3	5	ventral thalamus	-0.0152	1
wnt3	5	tectum cell layers	-0.164	0.2571
wnt3	5	telencephalon	0.1534	1
wnt3a	5	cerebellum ventricle	-0.025	1
wnt3a	5	diencephalon ventricle	-0.2722	0.0053
wnt3a	5	tectum midline ventricle	-0.5246	0.0018
wnt3a	5	tectum proliferative ventricle	-0.2722	0.2369
wnt3a	5	telencephalon ventricle	-0.1839	0.5977
wnt3a	5	cerebellum	-0.1432	1
wnt3a	5	dorsal thalamus	-0.2644	0.5168
wnt3a	5	pretectum	-0.3325	0.1087
wnt3a	5	ventral thalamus	-0.2719	1
wnt3a	5	tectum cell layers	-0.3556	0.0992
wnt3a	5	telencephalon	-0.0967	1

Table S3. Dysregulation of progenitor markers in areas lacking retinal input.

Table of markers for critical transitions during neuronal differentiation. Marker expression was quantified in key brain areas that receive retinal input and some that do not. Expression fold change shows whether a gene is up- or down-regulated in lakritz mutants. The p-values were calculated using a two-sided Wilcoxon signed-rank test and corrected for multiple testing using the Bonferroni correction.

Table S4

gene	age (dpf)	anatomical mask	expression fold change (log2)	adjusted p-value
neurog1	3	cerebellum ventricle	0.4084	0.3038
neurog1	3	diencephalon ventricle	0.0257	1
neurog1	3	tectum midline ventricle	0.2366	0.2258
neurog1	3	tectum proliferative ventricle	0.2263	1
neurog1	3	telencephalon ventricle	0.1914	1
neurog1	3	cerebellum	0.5399	0.0862
neurog1	3	dorsal thalamus	0.1889	0.3509
neurog1	3	pretectum	0.2975	0.0165
neurog1	3	ventral thalamus	0.4392	0.0511
neurog1	3	tectum cell layers	0.2625	0.2623
neurog1	3	telencephalon	0.1648	1
neurog1	4	cerebellum ventricle	0.0176	1
neurog1	4	diencephalon ventricle	-0.0626	1
neurog1	4	tectum midline ventricle	-0.103	1
neurog1	4	tectum proliferative ventricle	-0.0719	1
neurog1	4	telencephalon ventricle	0.1125	1
neurog1	4	cerebellum	0.0819	1
neurog1	4	dorsal thalamus	-0.0685	1
neurog1	4	pretectum	-0.0902	1
neurog1	4	ventral thalamus	0.0952	1
neurog1	4	tectum cell layers	-0.1874	1
neurog1	4	telencephalon	0.0606	1
neurog1	5	cerebellum ventricle	0.4766	0.0181
neurog1	5	diencephalon ventricle	0.3271	0.9556
neurog1	5	tectum midline ventricle	0.1882	0.2377
neurog1	5	tectum proliferative ventricle	0.3252	0.6699
neurog1	5	telencephalon ventricle	0.5117	0.0957
neurog1	5	cerebellum	0.1569	1
neurog1	5	dorsal thalamus	0.1227	1
neurog1	5	pretectum	0.0683	1
neurog1	5	ventral thalamus	0.2692	0.0789
neurog1	5	tectum cell layers	0.0786	1
neurog1	5	telencephalon	0.0951	1
neurog1	6	cerebellum ventricle	0.1627	1
neurog1	6	diencephalon ventricle	-0.3174	0.2623
neurog1	6	tectum midline ventricle	0.0655	1
neurog1	6	tectum proliferative ventricle	0.0728	1
neurog1	6	telencephalon ventricle	-0.1074	1
neurog1	6	cerebellum	0.2455	1
neurog1	6	dorsal thalamus	-0.1207	1
neurog1	6	pretectum	-0.1016	1
neurog1	6	ventral thalamus	0.0382	1
neurog1	6	tectum cell layers	-0.0577	1
neurog1	6	telencephalon	0.3123	0.2258
neurog1	7	cerebellum ventricle	0.5477	0.0742
neurog1	7	diencephalon ventricle	0.168	0.7942
neurog1	7	tectum midline ventricle	0.1527	1
neurog1	7	tectum proliferative ventricle	0.6417	0.002
neurog1	7	telencephalon ventricle	0.4223	0.0617
neurog1	7	cerebellum	0.3865	0.7942
neurog1	7	dorsal thalamus	0.1486	0.21
neurog1	7	pretectum	0.1473	0.0153
neurog1	7	ventral thalamus	0.4663	0.0032
neurog1	7	tectum cell layers	0.2744	0.004
neurog1	7	telencephalon	0.2387	0.6943
sox2	3	cerebellum ventricle	0.0662	1
sox2	3	diencephalon ventricle	-0.0036	1
sox2	3	tectum midline ventricle	-0.0485	1
sox2	3	tectum proliferative ventricle	-0.0551	1
sox2	3	telencephalon ventricle	0.0923	1
sox2	3	cerebellum	0.2559	0.606
sox2	3	dorsal thalamus	-0.0114	1

sox2	3	pretectum	-0.0436	1
sox2	3	ventral thalamus	0.0557	1
sox2	3	tectum cell layers	-0.1172	1
sox2	3	telencephalon	0.125	1
sox2	4	cerebellum ventricle	0.1859	1
sox2	4	diencephalon ventricle	0.0864	1
sox2	4	tectum midline ventricle	0.0514	1
sox2	4	tectum proliferative ventricle	-0.1656	1
sox2	4	telencephalon ventricle	0.1748	0.9988
sox2	4	cerebellum	-0.0194	1
sox2	4	dorsal thalamus	-0.0252	1
sox2	4	pretectum	-0.0978	0.8853
sox2	4	ventral thalamus	0.2221	0.0426
sox2	4	tectum cell layers	-0.1227	0.606
sox2	4	telencephalon	0.0447	1
sox2	5	cerebellum ventricle	0.0779	1
sox2	5	diencephalon ventricle	0.158	0.6389
sox2	5	tectum midline ventricle	0.1187	1
sox2	5	tectum proliferative ventricle	0.0232	1
sox2	5	telencephalon ventricle	0.1669	1
sox2	5	cerebellum	-0.0011	1
sox2	5	dorsal thalamus	0.0361	1
sox2	5	pretectum	0.0601	1
sox2	5	ventral thalamus	0.2146	0.531
sox2	5	tectum cell layers	0.0719	1
sox2	5	telencephalon	0.1029	1
sox2	6	cerebellum ventricle	-0.1351	1
sox2	6	diencephalon ventricle	-0.263	0.0232
sox2	6	tectum midline ventricle	-0.3775	0.0421
sox2	6	tectum proliferative ventricle	-0.024	1
sox2	6	telencephalon ventricle	-0.1047	1
sox2	6	cerebellum	-0.0142	1
sox2	6	dorsal thalamus	-0.2344	0.0284
sox2	6	pretectum	-0.3648	0.0421
sox2	6	ventral thalamus	-0.1104	1
sox2	6	tectum cell layers	-0.2803	0.3923
sox2	6	telencephalon	-0.0474	1
sox2	7	cerebellum ventricle	0.05	1
sox2	7	diencephalon ventricle	-0.0173	1
sox2	7	tectum midline ventricle	-0.2698	0.4179
sox2	7	tectum proliferative ventricle	0.0301	1
sox2	7	telencephalon ventricle	0.0704	1
sox2	7	cerebellum	-0.0252	1
sox2	7	dorsal thalamus	-0.1422	0.3024
sox2	7	pretectum	-0.2465	0.1044
sox2	7	ventral thalamus	-0.0489	1
sox2	7	tectum cell layers	-0.0898	1
sox2	7	telencephalon	0.356	0.1044

Table S4. Late precursor differentiation markers show age-dependent dysregulation.

The expression of for markers for glutamatergic and GABAergic fate commitment, neurog1 and sox2, was tracked from age 3 dpf to 7 dpf. The p-values were calculated using a two-sided Wilcoxon signed-rank test and corrected for multiple testing using the Bonferroni correction (same as in Table S3). Table includes the values of Table S3.
