# Supplementary material for: Retina-derived signals control pace of neurogenesis in visual brain areas but not circuit assembly
Source: Nat Commun. 2023 Sep 27;14:6020. doi: 10.1038/s41467-023-40749-1 (PMC10533834; doi:10.1038/s41467-023-40749-1)
Supplement: Supplementary file 3 — Reporting Summary [file 41467_2023_40749_MOESM3_ESM.pdf]

nature portfolio

Corresponding author(s): Herwig Baier

Last updated by author(s): Aug 3, 2023

Reporting Summary

Nature Portfolio wishes to improve the reproducibility of the work that we publish. This form provides structure for consistency and transparency in reporting. For further information on Nature Portfolio policies, see our Editorial Policies and the Editorial Policy Checklist.

Statistics

For all statistical analyses, confirm that the following items are present in the figure legend, table legend, main text, or Methods section.

n/a	Confirmed	
☐	☒	The exact sample size (*n*) for each experimental group/condition, given as a discrete number and unit of measurement
☐	☒	A statement on whether measurements were taken from distinct samples or whether the same sample was measured repeatedly
☐	☒	The statistical test(s) used AND whether they are one- or two-sided<br>*Only common tests should be described solely by name; describe more complex techniques in the Methods section.*
☐	☒	A description of all covariates tested
☐	☒	A description of any assumptions or corrections, such as tests of normality and adjustment for multiple comparisons
☐	☒	A full description of the statistical parameters including central tendency (e.g. means) or other basic estimates (e.g. regression coefficient) AND variation (e.g. standard deviation) or associated estimates of uncertainty (e.g. confidence intervals)
☐	☒	For null hypothesis testing, the test statistic (e.g. *F*, *t*, *r*) with confidence intervals, effect sizes, degrees of freedom and *P* value noted<br>*Give P values as exact values whenever suitable.*
☒	☐	For Bayesian analysis, information on the choice of priors and Markov chain Monte Carlo settings
☒	☐	For hierarchical and complex designs, identification of the appropriate level for tests and full reporting of outcomes
☒	☐	Estimates of effect sizes (e.g. Cohen's *d*, Pearson's *r*), indicating how they were calculated

Our web collection on statistics for biologists contains articles on many of the points above.

Software and code

Policy information about availability of computer code

Data collection	All code used is publicly available and visibly stated in the manuscript.
Data analysis	All code is implemented from published tutorials or publicly available python and R packages. Cellranger software suite (version 3.1.0 10X Genomics), R (version 3.6), Python (version 3.7, Anaconda3), Seurat (R, version 3), Scanpy (Python, version 1.9)

For manuscripts utilizing custom algorithms or software that are central to the research but not yet described in published literature, software must be made available to editors and reviewers. We strongly encourage code deposition in a community repository (e.g. GitHub). See the Nature Portfolio guidelines for submitting code & software for further information.

Data

Policy information about availability of data

All manuscripts must include a data availability statement. This statement should provide the following information, where applicable:

- Accession codes, unique identifiers, or web links for publicly available datasets
- A description of any restrictions on data availability
- For clinical datasets or third party data, please ensure that the statement adheres to our policy

Source data are provided with this paper. Relevant data supporting the key findings of this study are its Supplementary Information files or from the corresponding author upon request. All raw and processed scRNA-seq datasets reported in this study have been deposited in NCBI's Gene Expression Omnibus (GEO) under the accession number GSE238240.

Human research participants

Policy information about studies involving human research participants and Sex and Gender in Research.

Reporting on sex and gender	N/A
Population characteristics	N/A
Recruitment	N/A
Ethics oversight	N/A

Note that full information on the approval of the study protocol must also be provided in the manuscript.

Field-specific reporting

Please select the one below that is the best fit for your research. If you are not sure, read the appropriate sections before making your selection.

☒ Life sciences ☐ Behavioural & social sciences ☐ Ecological, evolutionary & environmental sciences

For a reference copy of the document with all sections, see nature.com/documents/nr-reporting-summary-flat.pdf

Life sciences study design

All studies must disclose on these points even when the disclosure is negative.

Sample size	For sequencing runs, the number of larvae used was determined by the maximum possible cell yield within the time limit ensuring healthy cells. For optogenetics experiments sample size was not calculated.
Data exclusions	Low-quality sequenced data were excluded by default set parameters. Low quality cells were excluded by thresholding mitochondrial percentage, as is common practice in the field.
Replication	For single-cell sequencing, 3 or 4 replicates were performed with multiple lanes sequenced for each replicate. This has been shown before to be sufficient to control for confounding batch and other technical effects. All attempts at replication were successful.
Randomization	No attempts to randomize test subjects were made as each group needed to be processed separately. For some statistical tests, the samples were posthoc randomly shuffled with stripped labels to test the significance of an effect.
Blinding	Investigators were not blinded to the allocation of each group. During data collection, it was not possible to mask the groups as the collection of larvae required a choice of group before the experiment. During data analysis it was not possible to mask the group labels as we needed to directly compare the mutant group against both control groups.

Reporting for specific materials, systems and methods

We require information from authors about some types of materials, experimental systems and methods used in many studies. Here, indicate whether each material, system or method listed is relevant to your study. If you are not sure if a list item applies to your research, read the appropriate section before selecting a response.

Materials & experimental systems

n/a	Involved in the study	
☒	☐	Antibodies
☒	☐	Eukaryotic cell lines
☒	☐	Palaeontology and archaeology
☐	☒	Animals and other organisms
☒	☐	Clinical data
☒	☐	Dual use research of concern

Methods

n/a	Involved in the study	
☒	☐	ChIP-seq
☒	☐	Flow cytometry
☒	☐	MRI-based neuroimaging

Animals and other research organisms

Policy information about studies involving animals; ARRIVE guidelines recommended for reporting animal research, and Sex and Gender in Research

Laboratory animals	Zebrafish larvae at 5-7 days post fertilization.
Wild animals	No wild animals were used in the study.
Reporting on sex	sex-based analysis was not relevant to the study as zebrafish do not display sexual differences at the larval stage.
Field-collected samples	No field collected samples were used in the study.
Ethics oversight	All animal procedures conformed to the institutional guidelines set by the Max Planck Society, with an animal protocol approved by the regional government (Regierung von Oberbayern).

Note that full information on the approval of the study protocol must also be provided in the manuscript.
